# Characterization of genomic diversity in bacteriophages infecting *Rhodococcus*

**DOI:** 10.1371/journal.pone.0352686

**Published:** 2026-06-29

**Authors:** Dominic R. Garza, Daria L. Di Blasi, Karen K. Klyczek, James A. Bruns, Randall J. DeJong, Ann M. Findley, Deborah Jacobs-Sera, Ana E. Garcia-Vedrenne, Sally Molloy, Colin M. Lewis, Isabel Light, Brianna Empson, Maisam Ghannam, Jorge Alfred Bonilla, Steven G. Cresawn, Rebecca A. Garlena, Daniel A. Russell, Graham F. Hatfull, Amanda C. Freise

**Affiliations:** 1 Department of Microbiology, Immunology, and Molecular Genetics, University of California, Los Angeles, California, United States of America; 2 Department of Biology, University of Wisconsin-River Falls, River Falls, Wisconsin, United States of America; 3 Department of Biology, Calvin University, Grand Rapids, Michigan, United States of America; 4 Department of Biology, School of Sciences, University of Louisiana at Monroe, Monroe, Louisiana, United States of America; 5 Department of Biological Sciences, University of Pittsburgh, Pittsburgh, Pennsylvania, United States of America; 6 Department of Molecular and Biomedical Sciences, University of Maine, Orono, Maine, United States of America; 7 Department of Biology, James Madison University, Harrisonburg, Viginia, United States of America; Universidad de Valparaiso, CHILE

## Abstract

Bacteriophages are ubiquitous and highly genetically diverse biological entities. Here we describe the isolation and bioinformatic characterization of 56 phages isolated on two *Rhodococcus* spp. They include both lytic and temperate phages and are grouped with previously described *Rhodococcus* phages into six clusters and 16 singletons based on genome similarity. Their genome sizes range from 43.9 kbp to 142 kbp and they have a G + C content ranging from 41.2% to 68.4%. Some of the *Rhodococcus* phages are more closely related to phages isolated on non-*Rhodococcus* Actinobacteria hosts than they are to phages isolated from the same host genus, demonstrating complex evolutionary histories. This study further expands the growing field of Actinobacteriophage genomics.

## Introduction

Bacteriophages infecting bacterial hosts in the phylum Actinobacteria exhibit an expansive spectrum of diversity [1]. Genomic analyses have been conducted on large numbers of phages isolated on a single host genus, such as *Mycobacterium [*2*]*, *Gordonia [*3*]*, *Arthrobacter [*4*]*, and *Microbacterium [*5*]*, facilitated by students and faculty in the in the Science Education Alliance-Phage Hunters Advancing Genomics and Evolutionary Science (SEA-PHAGES) and Phage Hunting Integrating Research and Education (PHIRE) programs [6]. These studies revealed that populations of phages infecting a single host include those that are genomically very similar and can be grouped into clusters and sub-clusters based on nucleotide sequence similarity and shared gene content, and others that are distinct enough to remain as ‘singleton’ phages [7,8]. The phage genomes are pervasively mosaic, and they share genes both within and between clusters. This mosaicism likely arises by horizontal gene transfer and illegitimate recombination as phages migrate across a landscape of bacterial hosts and acquire new genes from other phages and bacteria [9]. Analysis of phages from additional Actinobacteria strains will help to elucidate these mechanisms.

Comparative genome analyses of actinobacteriophages are facilitated by construction of databases of annotated genomes and grouping predicted protein coding genes into ‘phamilies’ (phams) according to amino sequence relationships of the predicted proteins [7,10]. Genes with no close relatives within this database are referred to as ‘orphams’ [7]. Pham assignments are made by the program ‘Phamerator’ which also constructs genome maps illustrating genome organization, pairwise nucleotide comparisons, and pham assignments [7]. Parameters for the phamily assignments, map constructions, and other bioinformatic considerations have been previously reported [8,11,12].

The role of *Rhodococcus* species in bioproduction, bioremediation and disease highlight the need for a better understanding of *Rhodococcus* phages. The *Rhodococcus* genus comprises Gram-positive Actinobacteria containing mycolic acids in their cell wall [13] and are commonly found in soil and aquatic environments [14]. *Rhodococcus* strains have played critical roles as biocatalysts in the synthesis of several organic compounds such as acrylamide, used in the production of polyacrylamide, and applications such as oil recovery and water treatment [15]. Additionally, there has been some success in the application of *Rhodococcus* for bioremediation efforts in contaminated environments due to their ability to break down polycyclic aromatic compounds [16]. There are two known pathogenic species of *Rhodococcus*; *R. fascians* is the causative agent of leafy gall disease in herbaceous perennials such as dahlias, petunias, and hostas, and *R. equi* (also known as *Prescottella equi*) [17] is an animal pathogen, causing pneumonia in young horses, immunocompromised horses, and immunocompromised humans [18]. *Rhodococcus erythropolis* was used as a bacterial host for phage discovery because it has simple biosafety precautions and is easy to grow and use for phage propagation.

Here, we describe the isolation and characterization of 57 phages isolated on *R. erythropolis* or *R. equi* and their comparative analysis with fourteen phages previously isolated on *Rhodococcus* species (Table 1) [19–26]. We describe the physical and genomic characteristics of the *Rhodococcus* phages, and the evolutionary relationships among these and other phages isolated on Actinobacteria hosts.

**Table 1:** *Rhodococcus* phages used in this study.

Phage Name	Cluster^1^	Isolation Host	Length (bp)	G + C%	ORFs	tRNAs	Accession #	Reference
Alatin	CA	*R. erythropolis* RIA 643	46673	58.7	68	3	MF324905	[29]
Alpacados	CA	*R. erythropolis* RIA 643	46493	58.9	68	2	MH271291	This study
AngryOrchard	CA	*R. erythropolis* RIA 643	46597	58.8	67	2	KY549153	[29]
AppleCloud	CA	*R. erythropolis* RIA 643	46389	58.7	66	2	MF324903	[29]
Belenaria	CA	*R. erythropolis* RIA 643	46538	58.6	71	3	MK524495	This study
BobbyDazzler	CA	*R. erythropolis* RIA 643	46641	58.8	68	3	KY549154	[29]
Bonanza	CA	*R. erythropolis* RIA 643	46932	58.8	69	3	MF537628	[29]
Bradshaw	CA	*R. erythropolis* RIA 643	46606	58.6	68	3	MH271293	This study
Bryce	CA	*R. erythropolis* RIA 643	46347	58.8	68	3	MH271294	This study
CosmicSans	CA	*R. erythropolis* RIA 643	46596	58.5	69	3	KT372002	[29]
Dinger	CA	*R. erythropolis* RIA 643	46617	58.8	69	3	MN945902	This study
Erik	CA	*R. erythropolis* RIA 643	46429	58.5	69	3	MH271297	This study
Espica	CA	*R. erythropolis* RIA 643	46537	58.6	71	3	MK524487	This study
Gollum	CA	*R. erythropolis* RIA 643	46535	58.6	69	3	MH271299	This study
Harlequin	CA	*R. erythropolis* RIA 643	46383	58.8	69	3	KX611788	[29]
Hiro	CA	*R. erythropolis* RIA 643	46854	58.7	68	3	MF324898	[29]
Jester	CA	*R. erythropolis* RIA 643	46314	58.7	68	3	MF373842	[29]
Krishelle	CA	*R. erythropolis* RIA 643	46985	58.5	70	3	MF324902	[29]
Lillie	CA	*R. erythropolis* RIA 643	46596	58.6	69	3	KT990218	[29]
Naiad	CA	*R. erythropolis* RIA 643	46619	58.6	68	3	MF324901	[29]
Nancinator	CA	*R. erythropolis* RIA 643	45936	58.6	68	3	MH271306	This study
Natosaleda	CA	*R. erythropolis* RIA 643	46527	58.6	68	3	KX550082	[29]
Partridge	CA	*R. erythropolis* RIA 643	46962	58.8	69	3	KX712237	[29]
PhailMary	CA	*R. erythropolis* NRRL B-1574	45614	58.9	65	2	MW291027	This study
Phrankenstein	CA	*R. erythropolis* RIA 643	46540	58.6	69	3	MH271309	This study
Rasputin	CA	*R. erythropolis* RIA 643	46568	58.8	69	3	MH271311	This study
RexFury	CA	*R. erythropolis* RIA 643	46627	58.6	68	3	MF324904	[29]
Rhodalysa	CA	*R. erythropolis* RIA 643	46596	58.5	69	3	KT375356	[29]
Shuman	CA	*R. erythropolis* NRRL B-1574	46544	58.6	70	3	MH316569	This study
StCroix	CA	*R. erythropolis* RIA 643	46619	58.6	68	3	MF324900	[29]
Swann	CA	*R. erythropolis* RIA 643	46596	58.6	69	3	MH271314	This study
Takoda	CA	*R. erythropolis* RIA 643	46807	58.7	70	3	MH271315	This study
TWAMP	CA	*R. erythropolis* RIA 643	46596	58.5	69	3	KT959213	[29]
UhSalsa	CA	*R. erythropolis* RIA 643	46539	58.6	69	3	MH271319	This study
Yogi	CA	*R. erythropolis* RIA 643	46930	58.8	69	3	KX712236	[29]
Yoncess	CA	*R. erythropolis* RIA 643	46353	58.8	68	3	MF189179	This study
RER2	CA	*R. erythropolis* Rery29	46596	58.6	67	3	JN116827	[21]
RGL3	CA	*R. globerulus* Rglo35	48072	62.7	66	3	JN116826	[21]
Grayson	CB	*R. erythropolis* RIA 643	131801	41.2	290	15	MH153812	This study
Peregrin	CB	*R. erythropolis* RIA 643	133006	41.4	287	14	MH153807	This study
Weasels2	CB	*R. erythropolis* RIA 643	134973	41.3	293	11	KX774321	This study
Pepy6	CC	*R. equi* 05–306	76797	53.4	107	1	GU580941	[26]
Poco6	CC	*R. equi* MillB	78064	53.3	107	1	GU580942	[26]
NiceHouse	CE	*R. erythropolis* NRRL B-1574	142586	44.9	291	31	MT521992	This study
Trina	CE	*R. erythropolis* RIA 643	139262	44.7	285	33	MF668286	This study
Jflix2	CF	*R. equi* NRRL B-16538	62017	66.5	104	0	OQ938594	[36]
Shagrat	CF	*R. equi* NRRL B-16538	62204	63.9	104	0	OQ938576	[36]
REQ1	CF	*R. equi* Requ28	51342	66.3	85	0	JN116825	[22]
Apiary	CR6	*R.* equi NRRL B-16538	67424	67.4	94	0	OR434026	[36]
Braxoaddie	CR6	*R. equi* NRRL B-16538	67415	67.3	97	0	OQ938585	[36]
Gagieri	CR6	*R. equi* NRRL B-16538	67084	67.1	93	0	OR434027	[36]
MacGully	CR7	*R. equi* NRRL B-16538	66998	66.2	102	0	OR283216	[36]
Maselop	CR6	*R. equi* NRRL B-16538	67419	67.3	93	0	OR475250	[36]
Polyyuki	CR6	*R. equi* NRRL B-16538	67447	67.3	94	0	OR475276	[36]
ChewyVIII	Sin	*R. erythropolis* RIA 643	69165	61.8	95	0	KX557288	This study
Finch	Sin	*R. erythropolis* RIA 643	138896	63.1	228	0	MG962366	This study
Jace	Sin	*R. erythropolis* RIA 643	53912	67	93	6	MH153804	This study
Reynauld	Sin	*R. equi* NRRL B-16538	80980	66.5	103	0	OR159659	[36]
Sleepyhead	Sin	*R. erythropolis* NRRL B-1574	43943	61	67	0	MK967380	This study
Trogglehumper	Sin	*R. erythropolis* NRRL B-1574	83592	63.8	121	0	OQ709222	This study
Whack	Sin	*R. erythropolis* NRRL B-1574	49660	61.9	77	0	MK967393	This study
DocB7	Sin	*R. equi* HDP1C	75772	56.7	105	0	GU580940	[26]
E3	Sin	*R. equi* NCIMB 10027	142563	67.5	209	0	HM114277	[23]
Pine5	Sin	*R. equi* 05–305	59231	67.1	84	0	GU580943	[26]
Mbo2	Sin	*R. opacus* PD630	83111	64.5	120	0	ON191531	N/A
Mbo4	Sin	*R. opacus* PD630	46226	65.2	66	0	ON191532	N/A
REQ2	Sin	*R. equi Requ28*	49330	65.4	82	0	JN116823	N/A
REQ3	Sin	*R. equi Requ28*	39474	65.9	60	0	JN116824	N/A
RRH1	Sin	*R. rhodochrous Rrho39*	14270	68.4	20	0	JN116822	[20]
Toil	Sin	*R. opacus* PD630	17253	54.5	35	0	KY817360	[19]

^1^Cluster or subcluster designation; Sin, Singleton.

## Results and discussion

### *Rhodococcus* phage isolation

Twenty-eight newly-isolated *Rhodococcus* phages were recovered from soil by plaque purification, using either enriched or direct methods. Most of these phages were isolated on *R. erythropolis* (47/56 phages); a small subset was isolated on *R. equi* (9/56 phages). Phages were isolated by students and faculty participating in the SEA-PHAGES or PHIRE program at 13 institutions (S1 Table). Comparative genomic analyses described here include an additional 42 Rhodococcus phages reported previously but not in the context of detailed comparative genomics. Table 1 lists the characteristics of all 70 *Rhodococcus* phages [20–24,26] and additional features of the phages are shown in S1 Table.

**Fig 1:**
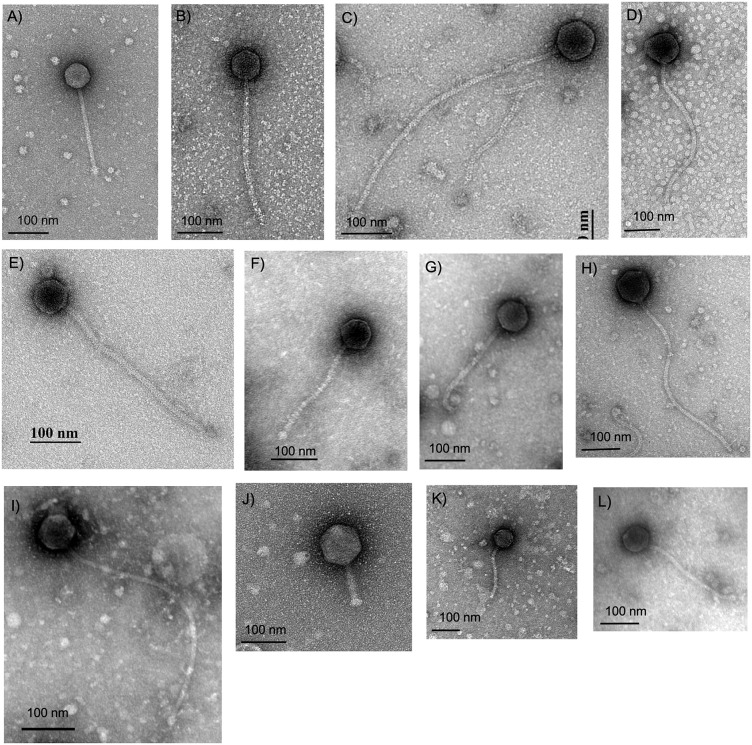
Transmission electron micrographs of *Rhodococcus* phages. Phages shown are: **A**) ChewyVIII, **B**) Sleepyhead, **C**) Trogglehumper, **D**) NiceHouse, (Cluster CE); **E**) Whack, **F**) Shagrat (Cluster CR6), **G**) MacGully (Cluster CR7), **H**) Weasels2 (Cluster CB), **I**) Reynauld, **J**) Finch, **K**) Rasputin (Cluster CA), and **L**) Maselop (Cluster CR6).

Transmission electron microscopy (Fig 1) shows that with only one exception all of the newly isolated *Rhodococcus* phages have siphoviral morphotypes, with isometric capsids and flexible non-contractile tails of varying lengths; the exception is Finch that has a myoviral morphology. Similarly, among the previously described *Rhodococcus* phages, E3 is a myovirus [24], Toil is a tectivirus (14), and all of the others are siphophages.

### *Rhodococcus* phage genometrics

Phage DNA was extracted, sequenced, and annotated using automated gene predictions followed by manual inspection and revision. GenBank accession numbers are shown in Table 1. Comparisons of the 56 newly isolated phages and 14 previously described phages showed that genome sizes range from 14,270 bp (RRH1) to 142,586 bp (NiceHouse) (Table 1). The genome termini vary in nature with most having short (5–11 bases) single-stranded 3’ extensions, but some are circularly permuted and presumably terminally redundant, and others have direct terminal repeats varying in length from 330 bp (Trogglehumper) to 5,291 bp (NiceHouse and Trina) (S1 Table). There is also substantial variation in G + C content (Table 1) and this is discussed in further detail below.

### Phage cluster assignments and relationships

**Fig 2:**
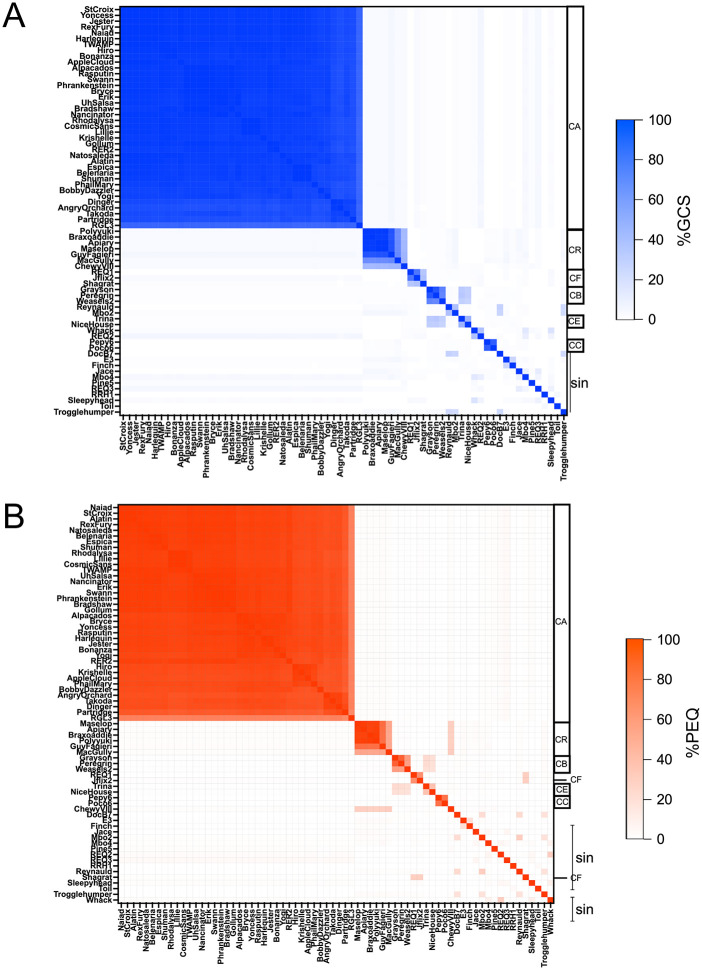
Genome comparisons of *Rhodococcus* phages. **A.** Heat map of the gene content similarity (GCS) among *Rhodococcus* phages. Pairwise GCS values between *Rhodococcus* phage genomes were computed and displayed in a heat map using Prism [27]. **B.** Heat map of proteomic equivalence quotient (PEQ) values of *Rhodococcus* phages. Pairwise PEQ values between *Rhodococcus* phage genomes were computed and displayed in a heat map using Prism [27].Clusters are denoted to the right; sin denotes singleton phages. Raw data are shown in S2 and S3 Tables.

The *Rhodococcus* phages were clustered using both gene content similarity (GCS) – with phages sharing approximately 35% or more of their genes assigned to the same cluster – and protein equivalency quotients (PEQ), both as described previously [3,10]. These analyses are in good agreement, and revealed six distinct clusters (CA, CB, CC, CE, CF, CR) and 16 singletons (Fig 2A, 2B; S2, S3 Tables). The only minor discrepancy is that the PEQ for Cluster CF phages Jflix2 and Shagrat is marginally below the 25% cutoff value (24.2), although the GCS is above 35% (S2 Table) justifying their grouping in Cluster CF. We note that the distribution of phages among the clusters is highly heterogenous, with Cluster CA being by far the largest with 38 members, Cluster CR with 6 and all other clusters with three or fewer members (Fig 2).

**Fig 3:**
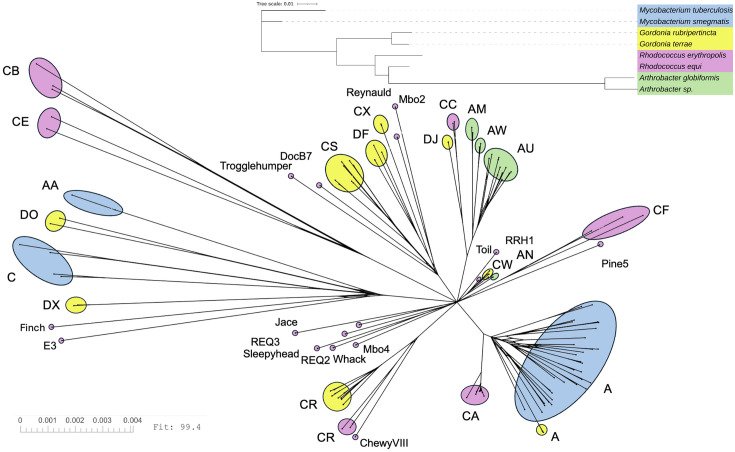
Gene content network phylogeny of *Rhodococcus* and related phages. SplitsTree6 was used to visualize phage relationships of clusters based on presence or absence of shared phams. Clusters are indicated by colored ovals with singletons labeled by phage name. Only selected clusters of *Mycobacterium*, *Gordonia*, and *Arthrobacter* phages are shown to emphasize the relationships. The color of the oval indicates the isolation host as specified in the legend. The scale bar indicates substitutions per site. Evolutionary relationships of bacterial host taxa inferred from 16S rRNA sequences. The 16S rRNA gene sequences from representative *Mycobacterium*, *Gordonia*, *Rhodococcus* and *Arthrobacter* hosts were aligned with MAFFT v7.525, trimmed using trimAl v1.5.0, and analyzed by maximum likelihood in IQ-TREE v3.0.1 under the TIM3 + F + I + G4 model selected by ModelFinder [28]. Node support was calculated from 1,000 ultrafast bootstrap and 1,000 SH-aLRT replicates. The resulting tree was imported into iTOL for final visualization and annotation.

In general, there is high intra-cluster pairwise shared gene content with a mean average value of 69.3%. Clusters CE (38.4% mean GCS) and CF (42.8% mean GCS) have among the lowest intra-cluster similarities (Fig 2A, 2B). Overall, the inter-cluster similarities are low (<1% GCS) (Fig 2A, 2B) indicating that these are not actively exchanging genes even though they infect the same or similar *Rhodococcus* hosts. These delineations are clearly reflected in a network phylogeny based on shared gene content (Fig 3; S4, S5 Tables). However, notable departures to this are the relatively close relationships between Cluster CB and CE phages (average 24.7% GCS), and between some of the singleton phages such as Whack and REQ2 (31.6% GCS), Reynauld and Trogglehumper (19.8%), Reynauld and Mbo2 (31.5% GCS), Reynauld and DocB7 (23.1% GCS), and Finch and E3 (20.7% GCS). ChewyVIII shares an average of 23.8% GCS with the Clusters CR6 and CR7. These are also reflected in their branch lengths in the network phylogeny (Fig 3).

The network phylogeny also reveals interesting relationships between the *Rhodococcus* phages and phages isolated on *Mycobacteria* spp, *Gordonia* spp, and *Arthrobacteria* spp. (Fig 3). The *Rhodococcus* Clusters CB, CE, and CF, and eight of the singletons (Toil, Jace, REQ3, Sleepyhead, REQ2, Whack, Pine5, and Mbo4) share few if any genes with these other phages (Fig 3). However, seven *Rhodococcus* phages cluster with 38 previously described *Gordonia* phages grouped in Cluster CR (Fig 3), although Cluster CR is very diverse and can be readily subdivided into seven subclusters. The *Gordonia* phages occupy Subclusters CR1 to CR5, whereas the *Rhodococcus* phages – all of which were isolated on *R. equi* NRRL B-16538 are in Subclusters CR6 and CR7 (Table 1, Fig 3) [3]. The only other Actinobacteriophage cluster containing phages isolated on more than one host is Cluster A, for which Subcluster A15 phages were isolated on *Gordonia* sp. and all others are mycobacteriophages [1]. It is notable though that Cluster CA *Rhodococcus* phages have substantial shared gene content with Cluster A phages (Fig 3).

Another notable set of relationships is between *Rhodococcus* singleton RHH1, the Cluster AN *Arthrobacter* phages, and Cluster CW *Gordonia* phages [3,4,20]. These are among the smallest Actinobacteriophage genomes (14–16 kbp) and include mostly virion structural genes with a small number of DNA-binding protein genes. We also note that *Rhodococcus* singletons Trogglehumper, DocB7, Reynauld, and Mbo2 share some genes with *Gordonia* phage (Clusters CS, DF, and CX), and singletons Finch and E3 share genes with *Gordonia* (Clusters DO and DX) and *Mycobacterium* phages (Clusters AA and C) (Fig 3). The *Rhodococcus* Cluster CC phages share genes with *Arthrobacter* (Clusters AM, AW, and AU) and *Gordonia* Cluster DJ phages as noted previously [4,5,26]. These phages share similar genome architectures including many small genes with transmembrane domains clustered in the center of the genome.

#### Phage lifestyles.

For many of the phages, plaque morphotypes do not unambiguously show if they are lytic or temperate. However, genomic characterization provides strong clues, as temperate phage often have recognizable integrase and repressor genes. Four clusters (CB, CC, CE and CR) and four newly isolated singletons (ChewVIII, Finch, Reynauld and Trogglehumper) are predicted to be lytic, and two (CA and CF) and three of the newly isolated singletons (Jace, Sleepyhead and Whack) are predicted to be temperate.

### Cluster-specific phage features

Each of the clusters and the singleton phages have notable features and are discussed in turn in the sections below.

#### Cluster CA.

**Fig 4:**
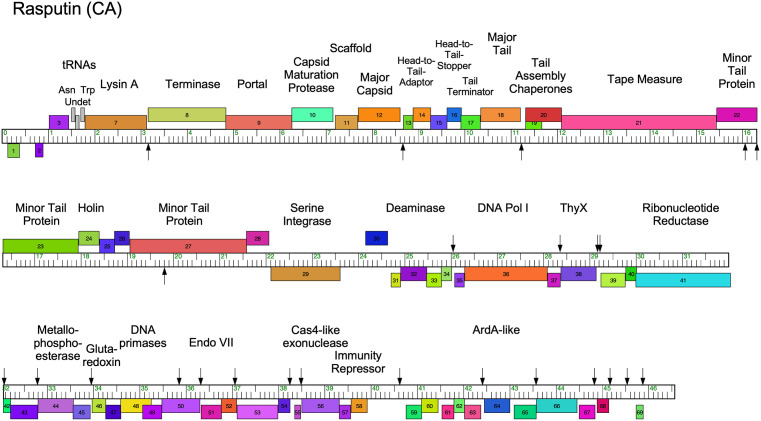
Genome organization of *Rhodococcus* Cluster CA phage Rasputin. The genome is shown with predicted genes represented as boxes above or below the genome reflecting rightwards and leftwards transcription, respectively. Genes are colored according to their phamily designations using Phamerator.org and database Actinobacteriophage_4268. Predicted gene functions are indicated above the genome. tRNA genes are represented by gray boxes with the amino acid specified above the box. Predicted gene functions are noted above the genes. Vertical arrows indicate the positions of putative stoperator sites with upwards and downwards arrows depict two different orientations of the asymmetric stoperators.

Cluster CA is the largest cluster of *Rhodococcus* phages, containing 38 of the 70 annotated phages. All Cluster CA phages were isolated on *R. erythropolis* except for the previously described phage RGL3, which was isolated on *R. globerulus* Rglo35 [20]. Cluster CA phages isolated on *R. erythropolis* share a high degree of sequence similarity; the entire cluster has a minimum pairwise GCS value of 75.8% (Fig 2) with a minor variations (S1A and S1B Figs). A detailed map of a representative Cluster CA phage (Rasputin) is shown in Fig 4.

Cluster CA phages have similarity to Cluster A phages and share closely related genome architectures [29]. Cluster CA phages share a mean GCS of 36.4% with phages in Cluster A, compared to a mean GCS of 1.3% with other *Rhodococcus* phages. The virion structural genes (e.g., terminase, portal protein, major capsid protein, capsid maturation protease, scaffolding protein, major tail protein, and head-to-tail connectors, tape measure protein) in the left arm are transcribed in the rightwards direction, while the right arm gene are leftwards-transcribed and encode DNA metabolism functions (e.g., DNA ph, DNA polymerase I, and ribonucleotide reductase; Fig 4). In an unusual departure in the genome organizations, the putative holin is not located near the lysin A gene (gene *7*; Fig 4) to the left of the terminase genes but is positioned among the minor tail protein genes (Fig 4). The identification of Rasputin 7 and its homologues as holins is supported by noting that a homolog in *Gordonia* Cluster CD phage Puppers is adjacent to the Lysin A gene, which is also near the minor tail protein genes.

Similarly to Cluster A, the Cluster CA phages are temperate [30], with a centrally located integrase gene of the serine-recombinase family (e.g., Rasputin *29*) and an immunity repressor gene (e.g., Rasputin *58*) in the genome right arm (Fig 4). It is not uncommon to find Cluster A phage that appear to be naturally lytic due to loss of the repressor gene, although this could arise from the selection of more visible plaques in the isolation process. This seems relatively rare among the Cluster CA phages, although Nancinator is one example. A notable feature of Cluster A phages are multiple copies of stoperator sequences (13–14 bp) located throughout the genome, positioned in one direction relative to transcription, and either intergenic or overlapping gene termini [31]. The Cluster CA phages share these features, and Rasputin (Fig 4) has 25 putative stoperator sites with the consensus sequence 5’-TGTCTATTGTCAAG. This differs from the consensus sequences in Cluster A phages [32,33].

Most of the Cluster CA phages code for at least two tRNA genes near the left ends of the genomes (Fig 4, S1 Fig). These tRNA-Asn and tRNA-Trp genes are separated by a region coding for a potential third tRNA whose charging potential is ambiguous; in some genomes at least one of the tRNA genes is absent or lost (S1 Fig). Transcriptomic analysis of Cluster CA phage WC1 during *R. erythropolis* infection [30] shows that these tRNA genes are transcribed late in phage infection. We note that in the previously described phages RER2 and RGL3 [21], the region corresponding to the left-most 2.5 kbp of the genome including the tRNA genes and the lysin A gene are located at the extreme right end of the genome. Although it is plausible that the *cos* site has been moved to a new location, it is also possible that the physical left ends of the viral genome are incorrectly identified.

#### Cluster CB.

**Fig 5:**
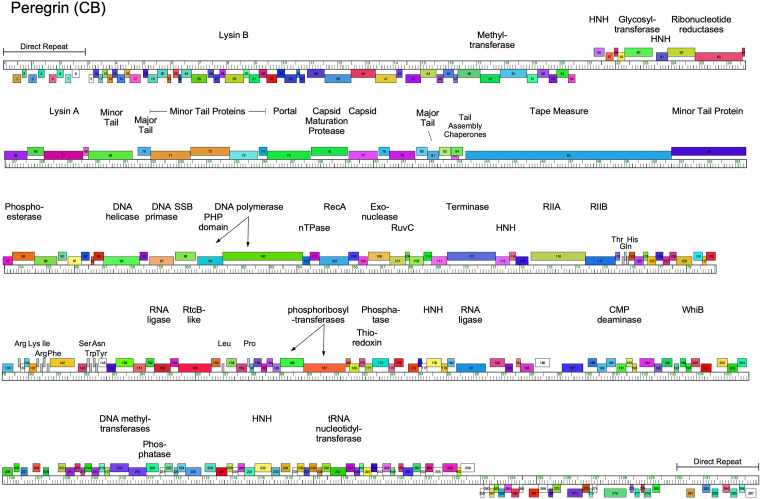
Genome organization of *Rhodococcus* Cluster CB phage Peregrin. See Fig 4 for details. White boxes indicate orphams (genes with no other phamily members).

The three Cluster CB phages (Grayson, Peregrin, and Weasels2) are lytic, and have unusually large genomes for siphophages, averaging 133 kbp (Table 1) and 277 predicted genes (Fig 5). The genome ends have long direct terminal repeats (2,900, 2,937, and 3,268 bp for Grayson, Peregrin, and Weasels2, respectively) each encoding eight putative genes (Fig 5). As of November 2025, these three phages have the lowest G + C content (41%) among the 5,460 sequenced actinobacteriophages genomes (https://phagesdb.org). Greyson and Peregrin are more similar to each other (85.0% GCS) than either phage is to Weasels2 (62.4% and 60.6% GCS, respectively; Fig 2, S2 Fig). The genome organization of Peregrin is shown in Fig 5.

The Cluster CB genomes have a different gene organization compared to other siphoviral phages (Fig 5). Most of the genes are rightwards-transcribed, but 55 putative genes at the left end of the genome and 29 at the right end are leftwards-transcribed. The virion structural genes have a non-canonical order, in which many of the minor tail protein genes (*69*–*74*) are positioned upstream of the portal-capsid genes (Fig 5). Additionally, the large terminase subunit (*112*) is located downstream and over 16 kbp away from the other virion structural genes. Oddly, there are two related putative major tail subunits in Peregrin (*70, 81*) and Grayson (*72, 83*) sharing 52% amino acid identity (Fig 5), and three in Weasels2 (*70, 72, 83*). It is not known if all of these are present in virions. These phages also code for a glycosyltransferase and a methyltransferase (Peregrin *60* and *49*, respectively) suggesting that the virions may be extensively glycosylated as reported for some other actinobacteriophages [34]. Two putative lysin genes (Lysin A and Lysin B) are also in unusual genomic locations (Fig 5). The genomes also code for several DNA metabolism functions including a DNA Pol III alpha subunit (Peregrin *100*).

The Cluster CB phages code for 11–15 tRNA genes (Table 1), mostly organized into several small clusters near the center of the genomes (Fig 5). Nearby are two genes coding for RNA ligase (Peregrin *153* and *155*), the latter of which is related to RtcB proteins, and these together with the tRNA genes may be involved in countering host defenses involving tRNA turnover. Peregrin and Weasels2 also encode a T4 rII-like system (Peregrin genes *116, 117)* [35], which is also implicated in interfering with defense systems.

Another striking feature of the Cluster CB phages is the abundance of small open reading frames with no known function; 72.8% of the Peregrin genes are shorter than 500 bp (Fig 5). This contributes to a relatively large number of genes compared to the genome size (Peregrin has 287 genes in its ~ 133 kbp genome). Weasels2, which is more distantly related to Peregrin and Grayson has an unusually large proportion (~30%) of Orphams [7] (S2 Fig), gene present only once on all of the actinobacteriophages (as of November 2025).

#### Cluster CE.

**Fig 6:**
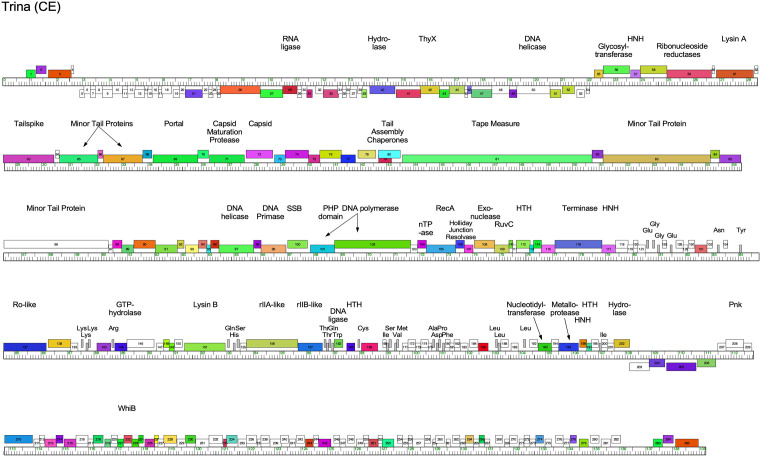
Genome organization of *Rhodococcus* Cluster CE phage Trina. See Figs 4 and 5 for details.

Cluster CE includes virulent phages NiceHouse and Trina, both of which were isolated on *R. erythropolis* NRRL B-1574. However, Trina and Nicehouse are not closely related (38.4% GCS) and only barely surpass the threshold for cluster inclusion (35% GCS). They are not closely related to the CB phages (Fig 2) but share many of the same features. They have large genomes for siphophages (~140–145 kbp) and a large number of genes (~290), many of which are small and of unknown function (Fig 6, S3 Fig, Table 1). They also share an unusual virion structure gene organization but have only a single major tail subunit gene. They code for many tRNA genes (33 in Trina and 31 in NiceHouse) as well as an RNA ligase and also have the T4 rII-like system and code for a DNA pol II alpha subunit, as in the Cluster CB phages. Both Trina and Nicehouse have an abundance of orphams (50.4% and 46.5% in Tina and NiceHouse, respectively).

#### Cluster CF.

**Fig 7:**
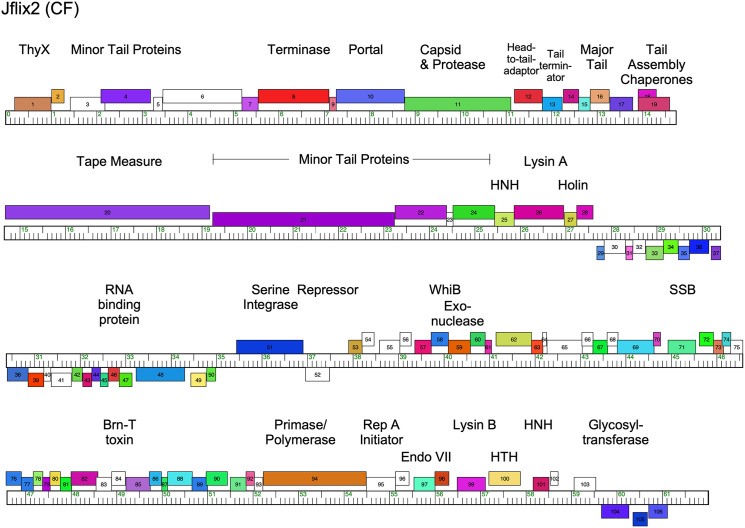
Genome organization of *Rhodococcus* Cluster CF phage Jflix2. See Figs 4 and 5 for details.

Cluster CF includes three temperate phages, Jflix2, Shagrat, and REQ1. Jflix2 and Shagrat were isolated on *R. equi* NRRL B-16538, and REQ1 reported previously [22] was isolated on *R. equi* Requ28. Although the PEQ values warrant cluster inclusion, some pairwise GCS values, such as between Jflix2 and Shagrat are relatively low (31.2%) (Fig 2, S4 Fig); a genome map of Jflix is shown in Fig 7. The structural gene organization is near-canonical for siphophages, although a tail protein is located near the left genome end as seen in some Cluster A phages. Interestingly, the capsid and capsid maturation protease functions are within a single gene (Jflix2 gene *11*; Fig 7). Cluster CF genomes code for a serine integrase (Jflix *51*) and an immunity repressor (Jflix *52*), consistent with them being temperate [36]. Shagrat and Jflix2 have a lysin A (Shagrat *27* and Jflix2 *26*). Host range analyses showed that Jflix2 and Shagrat did not form plaques on two additional *Rhodococcus* species (*R. globerulus* ATCC 15903, *R. jialingiae* ATCC 31636) or on two *Gordonia* species (*G. rubripertincta* ATCC 25593, *G. terrae* 3612) [36].

#### Cluster CR.

**Fig 8:**
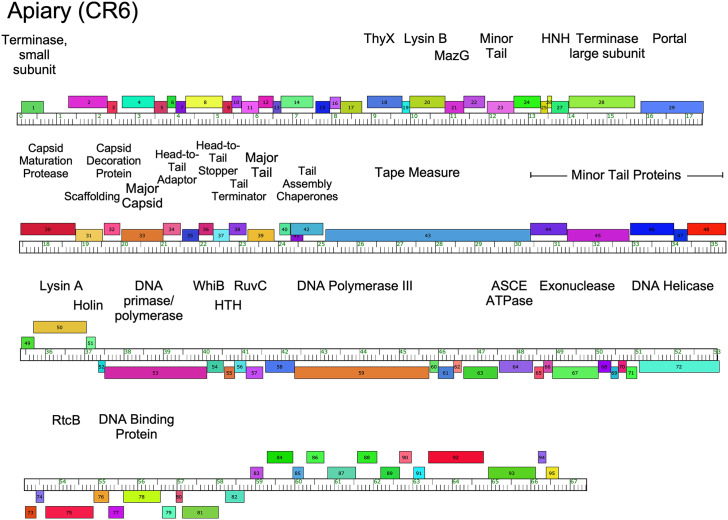
Genome organization of *Rhodococcus* Cluster CR phage Apiary. See Figs 4 and 5 for details.

Cluster CR phages are divided into seven subclusters (CR1 – CR7). However, the cluster contains phages isolated on both *Rhodococcus spp* and *Gordonia spp* and is divided into seven subclusters; all phages in Subclusters CR1 - CR5 were isolated on *Gordonia* and all of the phages in Subclusters CR6 and CR7 were isolated on *Rhodococcus* (Table 1). Nonetheless, the Cluster CR *Rhodococcus* phages do not infect *Gordonia* strains [36]. All Cluster CR phages have siphophage morphotypes and are predicted to be lytic. A map of Subcluster CR6 phage Apiary is shown in Fig 8, and the Cluster CR relationships are shown in S5 Fig.

Most of the Cluster CR phage genes are rightwards-transcribed (e.g., Apiary genes *1*–*50* and *83*–*95*), with a central segment of leftwards-transcribed genes (e.g., Apiary genes *52*–*82*) (Fig 8). The left-most series of rightwards-transcribed genes contains the virion structure and assembly genes, but also a block of genes – mostly of unknown function – between terminase small subunit gene (*1*) and the structural genes. Lysin A and holin genes are located to the right of the structural genes and the end of the putative late operon, although a gene related to mycobacteriophage Lysin B proteins is to the left the structural genes, and any role in lysis is unclear. The Cluster CR genomes do not encode tRNA’s, although they do code for an RtcB-like RNA ligase, and DNA metabolism functions including DNA Polymerase III alpha subunit, a primase/polymerase, a DNA helicase, and several putative regulators (Fig 8).

#### Singleton phages ChewyVIII, Finch, Jace, Sleepyhead, Whack, Reynauld, Trogglehumper.

**Fig 9:**
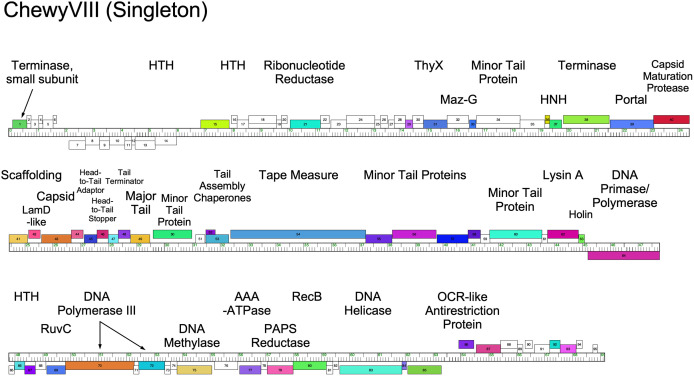
Genome organization of *Rhodococcus* singleton phage ChewyVIII. See Figs 4 and 5 for details.

ChewyVIII – isolated on *R. erythropolis* RIA 643 – is most closely related to Cluster CR phages (Fig 3); it shares a similar genome organization (Fig 9) but does not meet the GCS threshold criterion for cluster inclusion (S5Fig). It also includes an operon of leftwards-transcribed genes (*7*–*14*) near its left end that are absent from other Cluster CR phages (Fig 9). Moreover, about 50% of its genes are orphams with no closely related genes in the database used for comparisons (see Methods). ChewyVIII notably codes for an Ocr-like anti-restriction protein (ChewyVIII *86*) and homologues are present in Cluster BD *Streptomyces* phages (Fig 9).

**Fig 10:**
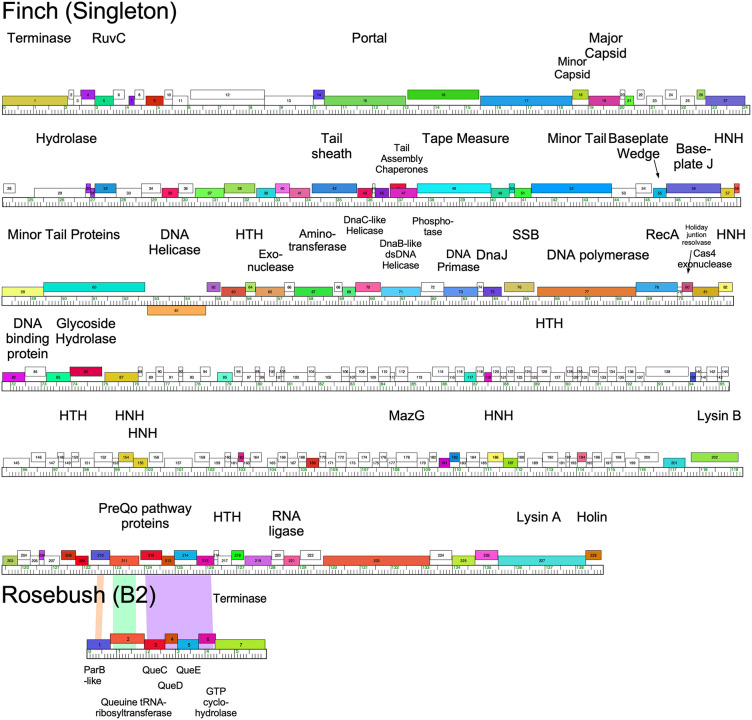
Genome organization of *Rhodococcus* singleton phage Finch. See Figs 4 and 5 for details. Also shown is the gene alignment with Mycobacterium phage Rosebush’s genes responsible for a PreQ_0_ pathway guanine modification [37].

*Rhodococcus* phage Finch and the previously described *Rhodococcus* phage E3 are the only *Rhodococcus* phages with myoviral morphology. Finch is predicted to be lytic and has a relatively large genome (138,896 bp), with circularly permuted and presumably terminally redundant genome termini (Table 1, S1Table, Fig 10). With the exception of gene *61* coding for a DNA helicase, all of the genes are rightward-transcribed (Fig 10). A striking feature of the genome is the large array of small genes (*88*–*200*) – mostly of unknown function – spanning ~40 kbp, most of which are orphams (Fig 10). Finch DNA is likely modified and is not sensitive to *Mse*I digestion (Fig 10); genes *210*–*215* are implicated in DNA modification because of their similarity to the DNA modification system in phage Rosebush as well as its relatives [37].

**Fig 11:**
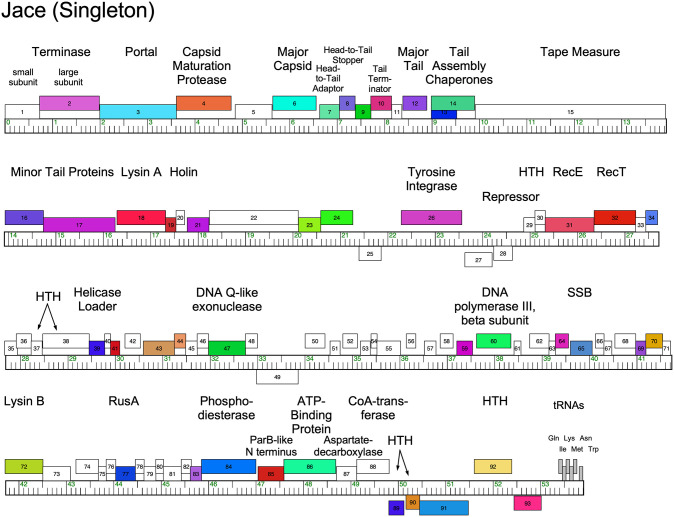
Genome organization of *Rhodococcus* singleton phage Jace. See Figs 4 and 5 for details.

Jace is a temperate siphophage sharing few genes with any other *Rhodococcus* phages (Fig 3). It is one of only two *Rhodococcus* phages with circularly permuted and presumably terminally redundant genomes, and the left was annotated as the first base of the small terminase subunit gene. Most of the genes are rightwards-transcribed, with the exceptions of genes *25, 27,28, 49, 89–91* and *93* (Fig 11). It also codes for six tRNAs, with the genes located at the extreme right end of the genome. As a temperate phage it codes for a putative repressor (gene *28*) with similarities to the immunity repressors of *Gordonia* Cluster DN phages, and a tyrosine-integrase (gene *26*) (Fig 11). A putative *attP* site is positioned immediately to the left of the integrase gene containing a region of 49 bp nucleotide identity with the 3’ end of a tRNA-gly gene in the *Rhodococcus* genome that defines the chromosomal *attB* site. Over half of the Jace genes are orphams with no homologues in the database used for comparisons (Fig 11).

**Fig 12:**
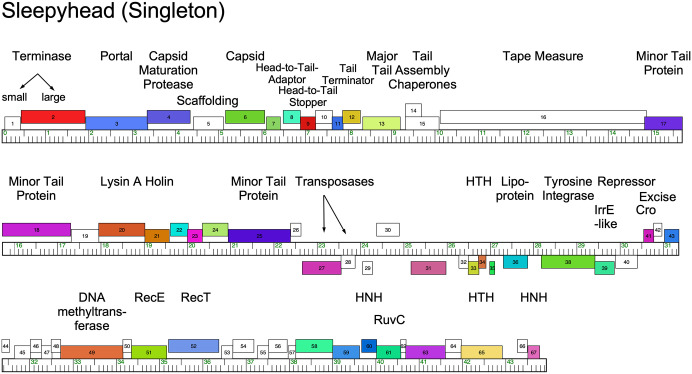
Genome organization of *Rhodococcus* singleton phage Sleepyhead. See Figs 4 and 5 for details.

Sleepyhead is a siphophage isolated on *R. erythropolis* NRRL B-1574 (Fig 12) and has 67 protein-coding genes but no tRNA genes (Table 1). The most closely related phage is *Rhodococcus* phage Whack, with which it shares 10 genes. Sleepyhead is predicted to be temperate, with the repressor and integrase encoded by genes *40* and *38*, respectively (Fig 12). The *attP* site is located immediately to the left of the *int* gene and interestingly has a 91 bp near perfect match (90/91) to the extreme 3’ end of an acyl co-A dehydrogenase family protein (gene QIE55_RS12330 in the *Rhodococcus erythropolis* MGMM8 genome (Accession # NZ_CP124545.1). Presumably Sleepyhead uses this as an *attB* site but notably reconstructs the 3’ end of the coding region to retain functionality. Sleepyhead forms turbid plaques on a lawn of *R. erythropolis* and forms stable lysogens that release phage particles into culture supernatant and are immune to superinfection (not shown). Sleepyhead carries an IS3 like transposon flanked by 13 bp inverted repeats (5’-GGGCCTTGACCCCG) flanked by a 3 bp target duplication. It codes for two open reading frames (*27, 28*) that are likely expressed as single polypeptide DDE transposase by a programmed translational frameshift (S6 Fig), as with other IS3-like elements [38]. Related transposons are present in other actinobacteriophages infecting other bacterial hosts including UncleRicky (*Mycobacterium*), and Blueberry, Whitney and Cucurbita (*Gordonia*) (Fig 12; S7 Fig).

**Fig 13:**
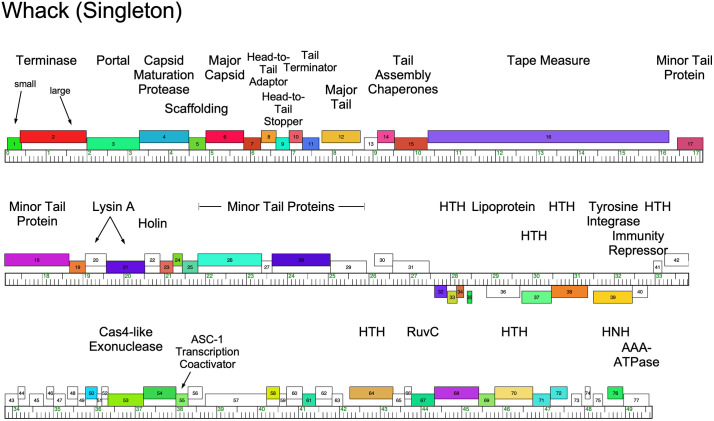
Genome organization of *Rhodococcus* singleton phage Whack. See Figs 4 and 5 for details.

Whack was isolated on *Rhodococcus erythropolis* NRRL B-1574 and is most closely related to the singleton REQ2, but sharing only 31.6% GCS, below the threshold for cluster inclusion (Fig 2, S8 Fig). It is predicted to be temperate (Fig 13). Most of the genes are transcribed rightwards (*1*–*31, 41*–*77*) but nine genes (*32–40*) are leftwards-transcribed including the putative repressor (*40*) and integrase (*39*) genes (Fig 13). The location of the *attP* is unclear, and it could be located in the short intergenic region to the left of the integrase gene, but there are no compelling sequence matches to *Rhodococcus* genomes that might indicate the potential *attB* site. The virion structural genes have a canonical organization for siphophages, although the lysis cassette appears to be unusually located within the minor tail protein genes (Fig 13).

**Fig 14:**
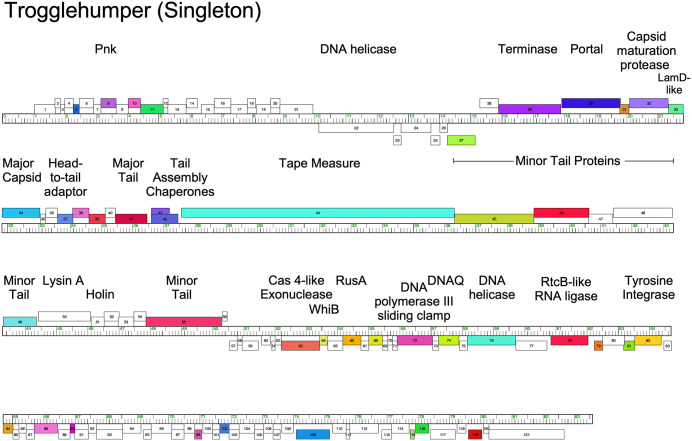
Genome organization of *Rhodococcus* singleton phage Trogglehumper. See Figs 4 and 5 for details.

**Fig 15:**
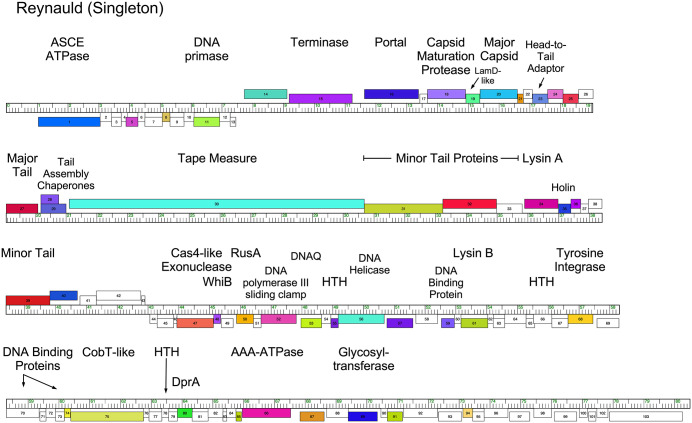
Genome organization of *Rhodococcus* singleton phage Reynauld. See Figs 4 and 5 for details.

Singleton phages Trogglehumper (Fig 14) and Reynauld (Fig 15) have similar virion morphologies with isometric heads and unusually long tails (Fig 1). They also have similar genome sizes and organizations and share some genes with each other (19.8% GCS), particularly among the virion structure and assembly genes. They are also genetically related to *Rhodococcus* singleton phages DocB7 and Mbo2 and *Gordonia* phages in Clusters CS, CX, and DF (Fig 4). Their genome structures are similar, with similar lengths and G + C contents (Table 1), direct terminal repeats of 330 and 362 bases (Reynauld and Trogglehumper, respectively) and a genome organization where most of the left arm is transcribed in the forward direction and the right arm is transcribed the reverse direction. It’s likely that all these phages are lytic, and immunity repressor genes were not identified. However, they all have genes coding for a tyrosine-recombinase situated amid a series of left-wards transcribed genes in the right parts of the genome (Figs 14, 15). It seems unlikely that these recombinases are used as phage integrases for prophage integration and are probably involved in unrelated recombination events.

#### Variations in GC content and codon usage.

**Fig 16:**
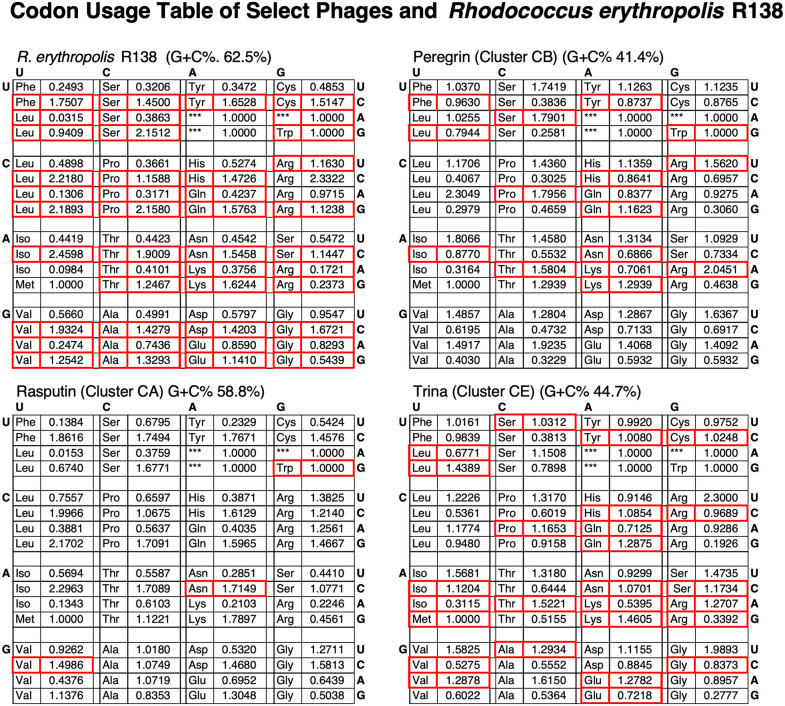
Codon usage table of *Rhodococcus* phages and their host. Display of codon usage using RSCU (Relative Synonymous Codon Usage) as calculated in DNA Master. The boxes outlined in red match the tRNAs of each phage and the host.

The *Rhodococcus* phages are unusual among the actinobacteriophages in having a wide range of GC contents, from 41.1% (Cluster CB phage Grayson) to 67.5% (singleton phage E3) and the Cluster CB and CE phages have some of the lowest GC of any of the actinobacteriophages. The *Rhodococcus* hosts have on average 62% G + C [39–42] such that there are substantial mismatches in G + C% content illustrated by Cluster CB phage Peregrin (41.4%) and its host *R. erythropolis* R138 (62.5%); this is even greater mismatch than between mycobacteriophage Patience (50.3% G + C) and its *M. smegmatis* host (65%) analyzed previously [43]. Not surprisingly, Peregrin has a very different profile of codon usage than *R. erythropolis* (Fig 16) including the predominance of codons with A or U in their third position relative to those with G or C (Fig 16). Because Peregrin codes for 14 tRNAs, this raises the question as to whether the tRNA specificities could explain the demand to translate codons that are typically rare in the host but abundant in the phage. However, this does not appear to be the case, as although Peregrin has a tRNA with an anticodon matching the rarest codon in the host (UUA), it also has tRNAs matching the related codon UUG which is very common in the host. Similarly, Peregrin has tRNAs with anticodons matching both of the lysine codons AAA and AAG, and in this instance the AAG codon is more common than AAA. We also note that in the host a single tRNA recognizes both of the tyrosine codons UAU and UAC, although the UAC codon is much more abundant (Fig 16).

Comparison of the Cluster CE phage Trina (44.7% G + C) codons usage with *R. erythropolis* shows a similar pattern. Again, the phage codons with A or U in their third position mostly outnumber those with C or G in each codon set (Fig 16). However, the tRNA profile is very different from that of Peregrin, and of the 33 Trina tRNAs, only 13 have similar anticodons to the Peregrin tRNAs. It is also notable that Trina has anticodons for Valine (codons GUN), Alanine (codons GCN), Glutamic Acid (codons GAR) and the Glycine codon GGC, all of which are absent from Peregrin (Fig 16). We note that some *Rhodococcus* phages (e.g., Cluster CA phage Rasputin) have tRNA genes but have similar G + C% to their bacterial hosts and similar codon usage profiles (Fig 16). For example, Rasputin has a tRNA-Trp although there is only a single tryptophan codon, and the other two tRNAs (tRNA-Asn and tRNA-Val) code have anticodons recognizing the codons with those codon sets that are the most abundant in the phage and the host.

These observations suggest that the role of the *Rhodococcus* phage-encoded tRNAs is not to compensate for the strong mismatches in codon usage that arise from substantial differences in GC. An alternative explanation is phages carry these tRNAs to cover host defenses that involve inactivation of tRNAs and abortive infection [44]. It is remarkable, however, that phages such as those in Cluster CE carry more than 30 tRNA genes, suggesting that the host defenses would involve widespread and non-specific tRNA destruction. The reason for the tRNA repertoire in phage genomes thus remains elusive and warrants further exploration.

### Concluding remarks

The newly isolated *Rhodococcus* phages described here expand our view of the diversity of these phages and their genetic repertoires. As seen with other actinobacteriophages including those isolated on *Mycobacterium*, the types of *Rhodococcus* phages are heterogenous, with the temperate Cluster CA phages being predominant and representing over half of those described here. In contrast, there are 16 singleton phages, showing that *Rhodococcus* phages are greatly under sampled, and more of these singletons as well as new singleton types have yet to be isolated. An additional feature shared with *Mycobacterium* phages is that only two virion morphotypes are seen, with only siphophages and myophages represented. The relative abundance of lytic and temperate phages among genomic types is not dissimilar to that found in *Mycobacterium* phages. It is notable that the most common *Mycobacterium* phages and *Rhodococcus* phage – in Clusters A and CA, respectively – are both temperate, which might suggest that they are integrated as prophages in related environmental strains.

The *Rhodococcus* phages have the broadest range of GC contents among any of the actinobacteriophages, resulting in those with lower GC contents having greatly different codon usage profiles than the host. We favor the explanation described previously for mycobacteriophage Patience that the lower GC phages have largely evolved in bacterial hosts with lower GC contents and only relatively recently acquired the ability to infect and replicate in their current hosts. Although it might be anticipated that acquisition of tRNA genes could help to compensate for relative paucity of host tRNAs for infrequently used codons in the host, that does not appear to occur. *Rhodococcus* phages with low GC do carry a large repertoire of tRNA genes, but these do not appear to exclusively compensate for coding deficiencies.

The *Rhodococcus* phage genomes raise many questions about their biology and replication processes, including host ranges and their determinants, the roles of the large number of genes unknown function, the action of the lysis systems, and life cycle regulation. The isolation and characterization of these newly isolated *Rhodococcus* phage will help to provide answers to these questions, as well as expanding our view of actinobacteriophage diversity.

## Materials and methods

### Bacterial strains & media

All phages were isolated on one of two different species of *Rhodococcus*: *R. equi* (NRRL strain B-16538) or *R. erythropolis* (NRRL strain B-1574 or RIA strain 643) (Table 1) [29,36,45]. PYCa media (containing per 1 liter volume: 1.0 g Yeast Extract, 15.0 g Peptone, 2.5 ml of 40% Dextrose, 2.5 ml of 1 M CaCl_2_, and 1 ml of 1000X CHX) or BD Difco nutrient broth with added agar to 1.6 or 0.45% for agar plates or top agar overlay was used for phage isolation, purification and amplification.

### Phage isolation, purification, amplification, and virion analysis

Phages were isolated from soil samples collected at several SEA-PHAGES institutions (S1 Table) using either an enriched or direct isolation protocol as described previously [29,36,45]. Multiple rounds of plaque picking and plaque assays were performed to ensure a homogenous phage population. Phage titers and plaque morphology were determined during each round of purification. High-titer phage lysates were generated by flooding plaque assay plates showing webbed lysis with phage buffer (containing per 1 liter volume: 10 ml of 1 M Tris stock (pH 7.5), 10 ml of 1 M MgSO_4_, 4 g NaCl, 10 ml of 100 mM CaCl_2_), incubating for at least 24 hrs at 4°C and then filtering through a 0.22 µm filter. Phage particles were imaged using transmission electron microscopy (TEM) where phage lysates were spotted onto carbon coated copper grids, stained with 1% uranyl acetate and imaged using a HITACHI 7800 TEM.

### Genome sequencing, assembly and annotation

Phage DNA was extracted using a Promega Wizard DNA Extraction kit or phenol chloroform, and phage genomes were sequenced at either the Pittsburgh Bacteriophage Institute or Western Carolina University using Illumina, Ion Torrent, or Roche 454 methods [46]. Raw reads were assembled with Newbler (GS De Novo Assembler version 2.9), and assemblies were checked for completeness, accuracy, and genomic termini using Consed [47]. Phage genomes were annotated as described previously [48] using DNA Master, GLIMMER [49], GeneMark [50], BLAST [51], Aragorn [52] and tRNAScan-SE [53]. Gene functions were determined using Blast, HHpred [54], the Conserved Domain Database (CDD) [55], TMHMM [56] and DeepTMHMM [57]. Genome comparisons were performed using Phamerator.org and database Actinobacteriophage_4268 (created December 8, 2023).

### Genome analyses

Phage genes were grouped into phamilies (phams) of closely related proteins using Phamerator via the pdm_utils database management package [7,12]. Genome architecture, and nucleotide sequence similarity were compared using Phamerator.org (https://phamerator.org/) [7]. Gene Content Similarity (GCS) analysis was performed with PhamClust using the ‘-m gcs’ flag, and data were visualized using GraphPad Prism (www.graphpad.com). PEQ values were calculated using PhamClust [10] and visualized using GraphPad Prism. For cross-host analyses, two representative phages were selected from each cluster with more than three members (S1 Table). Gene content network phylogenies were created using PhamNexus and SplitsTree6 [58] to render an unrooted tree using equal angle and Neighbornet functions. Intra-cluster pham matrix comparisons were performed using a custom code notebook on the Observable platform (https://observablehq.com/@cresawn-labs/pham-matrix). Codon usage biases were estimated using all genes from phage genomes Rasputin, Peregrin, Trina, and model host *Rhodococcus erythopolis* R138 (CP007255). Relative synonymous codon usage (RSCU), as part of the DNA Master program, is the ratio of the codon count to the amino acid count that was then used to normalize the codon frequencies so that the sum of RSCU for codons of each amino acid equals the number of synonymous codons for each amino acid. Actual codon counts are found in supplemental data (S6 Table).

## Supporting information

S1 Table Additional *Rhodococcus* phage information. (XLSX)

S2 Table Pairwise GCS data for all *Rhodococcus* phages. (XLSX)

S3 Table Pairwise PEQ values for all *Rhodococcus* phages. (XLSX)

S4 Table Pairwise GCS values for representative Rhodococcus and non-Rhodococcus actinobacteriophages. (XLSX)

S5 Table Pairwise PEQ values for representative Rhodococcus and non-Rhodococcus actinobacteriophages. (XLSX)

S6 Table Codon usage table counts. Relative Synonymous Codon Usage numbers and actual codon counts are displayed in table format for each phage or host listed.
(XLSX)

S1 Fig Genomic diversity of Cluster CA phages. **A)** Phamerator.org map. Each phage genome is shown with predicted genes represented as boxes above or below the genome reflecting rightwards and leftwards transcription, respectively. Each colored box represents a gene, colored according to pham membership as defined by Phamerator.org. White boxes are orphams (genes with no other phamily members). The shading between genomes indicates pairwise nucleotide identity in rainbow order, with purple indicating high similarity, red indicating low similarity, and white indicating no similarity**. B)** Pham matrix. Each row represents a phage gene map with rectangular boxes representing genes. All boxes are the same width, irrespective of the nucleotide length of the gene. The genes are color-coded by phamily as defined by Phamerator.org, with white boxes indicating orphams. Each column of boxes represents a gene phamily, arranged by mean genomic position along the X axis.
(PDF)

S2 Fig Genomic diversity of Cluster CB phages. See S1 Fig for details.
(PDF)

S3 Fig Genomic diversity of Cluster CE phages. See S1 Fig for details.
(PDF)

S4 Fig Genomic diversity of Cluster CF phages. See S1 Fig for details.
(PDF)

S5 Fig Genomic diversity of Cluster CR phages. See S1 Fig for details of **A)** and **B). C).** Display of Gene Content Similarities (GCS) amongst the Rhodococcus Cluster CR phages and singleton ChewyVIII.
(PDF)

S6 Fig The nucleotide sequence and three reverse oriented translational reading frames of the Sleepyhead insertion sequence. The direct repeats (TG) are indicated in bolded nucleotides and the left and right inverted repeats (LIR and RIR) are indicated by arrows. The gray arrows represent protein coding genes with gp28 (light gray) and gp27 (dark gray) as ORF 1 and ORF2 of the insertion sequence, respectively. The −10 and −35 boxes of a putative promoter overlapping the RIR is indicated by light green arrows and underlined nucleotides. The reading frames for Sleepyhead gp27 and 28 are highlighted in light gray. The TTTC tetramer at the 3’ end of ORF1 (gp28) that potentially signals a −1 programmed ribosomal frameshift is highlighted in light blue.
(PDF)

S7 Fig Alignment of DDE transposase sequences. The DDE transposase (Pham 8239; in purple) is found in the genomes of *Rhodococcus* phage, Sleepyhead (Singleton), *Gordonia* phages Blueberry (CV), Whitney (DN1), Cucurbita (CQ1) and *Mycobacterium* phage UncleRicky (F1). Sleepyhead 27 encodes a DDE transposase and gene 28 has strong HHpred alignments to helix-turn-helix DNA binding domains associated with transposases (PF01710) [59]. The DDE transposase pham is found in the phage genomes of UncleRicky (Subcluster F1), Cucurbita (cluster CQ), Blueberry (Cluster CV), and Whitney (Cluster DN1) (Fig 14). The insertion sequence in these phage genomes has a second ORF that belongs to a different pham than Sleepyhead gp28, however all these genes have the same HHpred alignments to transposase associated helix-turn-helix DNA binding domains. While the transposase of some insertion sequences is expressed from a single ORF, transposases of the IS3 family typically exist as two overlapping open reading frames with ORF2 being in the −1 frame relative to ORF1 [38]. These have a −1 programmed ribosomal frameshift in the overlapping region of ORF1 and ORF2 that is signaled by a X-XXZ-ZZN heptamer or a Z-ZZN tetramer [25,60]. A T-TTC tetramer exists just upstream of the Sleepyhead gp28 stop codon that would allow a −1 frameshift and translation of Sleepyhead gp28:27 fusion protein 408 amino acids long (S6 Fig) [61,62].
(PDF)

S8 Fig Genome comparison of phages Whack and REQ. Phamerator.org map. Each phage genome is shown with predicted genes represented as boxes above or below the genome reflecting rightwards and leftwards transcription, respectively. Each colored box represents a gene, colored according to pham membership as defined by Phamerator.org. White boxes are orphams (genes with no other phamily members). The shading between genomes indicates pairwise nucleotide identity in rainbow order, with purple indicating high similarity, red indicating low similarity, and white indicating no similarity.
(PDF)
