# Supplementary material for: Characterization of genomic diversity in bacteriophages infecting Rhodococcus
Source: PLoS One. 2026 Jun 29;21(6):e0352686. doi: 10.1371/journal.pone.0352686 (PMC13313380; doi:10.1371/journal.pone.0352686)
Supplement: S1 Fig — A) Phamerator.org map. Each phage genome is shown with predicted genes represented as boxes above or below the genome reflecting rightwards and leftwards transcription, respectively. Each colored box represents a gene, colored according to pham membership as defined by Phamerator.org. White boxes are orphams (genes with no other phamily members). The shading between genomes indicates pairwise nucleotide identity in rainbow order, with purple indicating high similarity, red indicating low similarity, and white indicating no similarity. B) Pham matrix. Each row represents a phage gene map with rectangular boxes representing genes. All boxes are the same width, irrespective of the nucleotide length of the gene. The genes are color-coded by phamily as defined by Phamerator.org, with white boxes indicating orphams. Each column of boxes represents a gene phamily, arranged by mean genomic position along the X axis. (PDF) [file pone.0352686.s007.pdf]

Genomic diversity of Cluster CA phages

A)

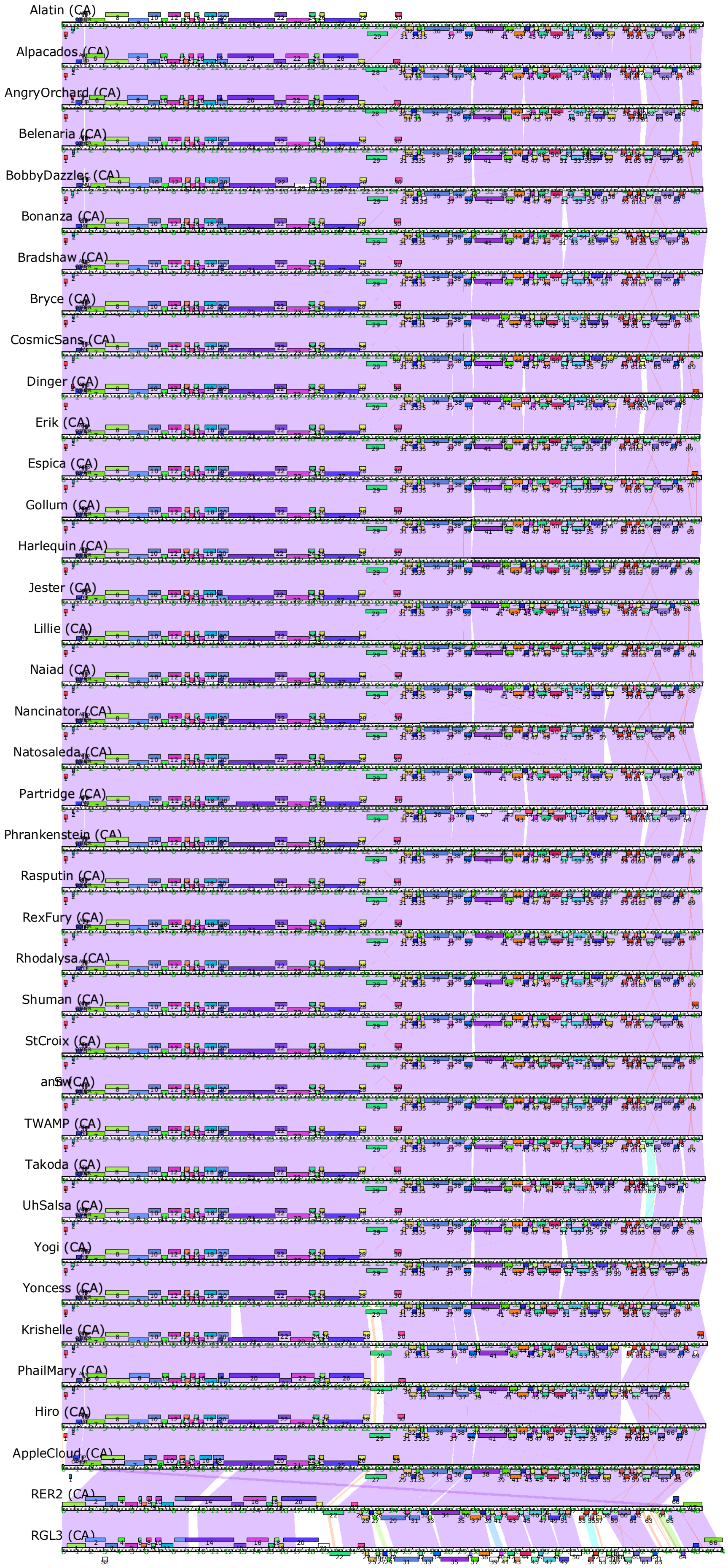

S1A_Figure

Genomic diversity of Cluster CB phages

B)

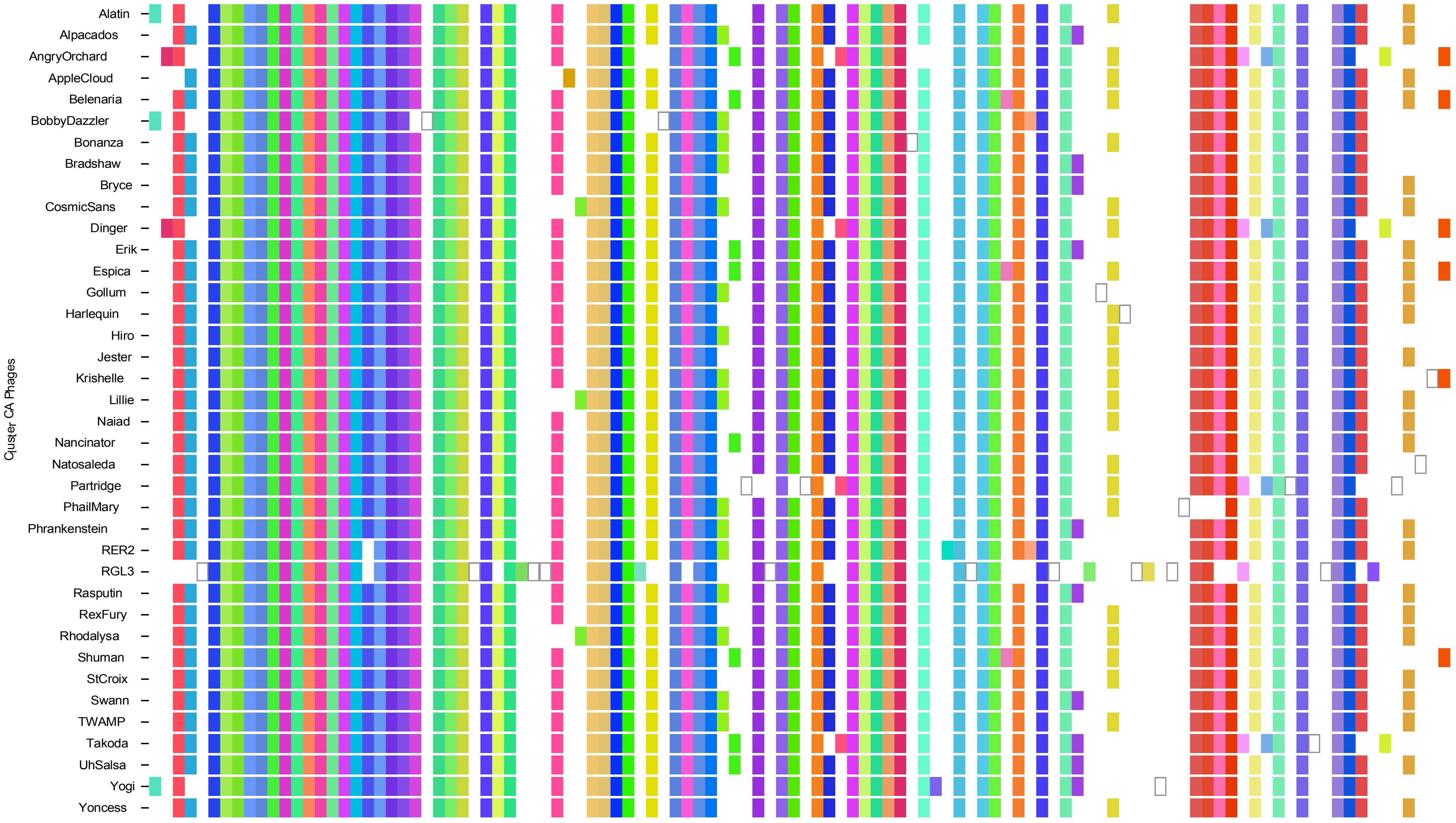

S1B_Figure
