# Supplementary figures and images for: Characterization of genomic diversity in bacteriophages infecting Rhodococcus
Source: PLoS One. 2026 Jun 29;21(6):e0352686. doi: 10.1371/journal.pone.0352686 (PMC13313380; doi:10.1371/journal.pone.0352686)

# Genomic diversity of Cluster CB phages

A)

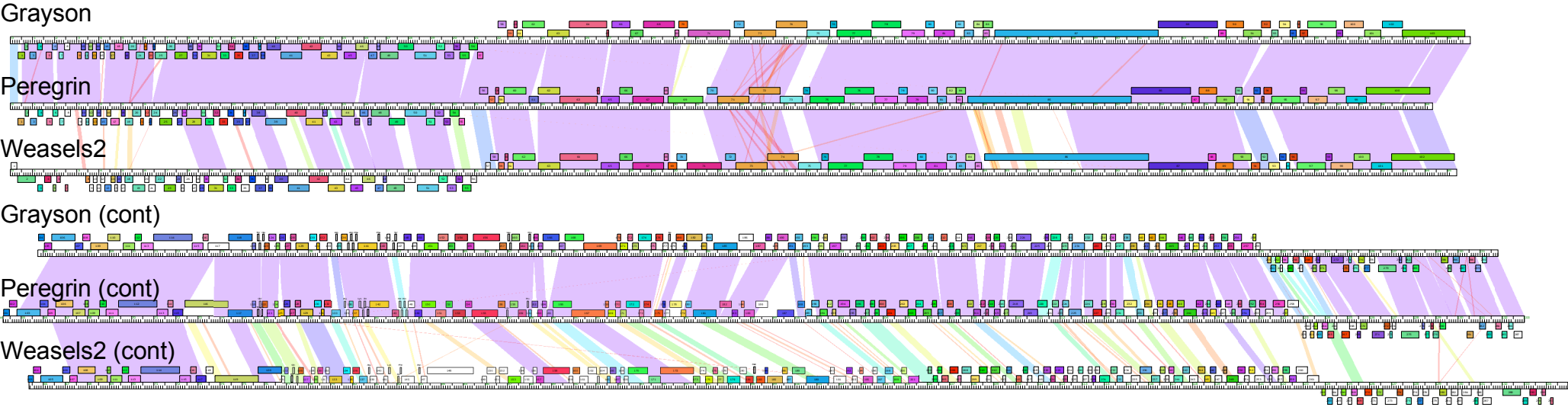

B)

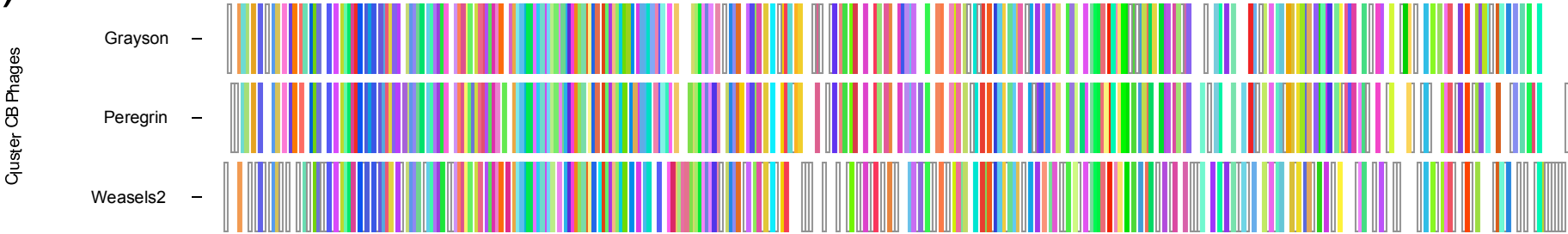

Supplement: S2 Fig — See S1 Fig for details. (PDF) [file pone.0352686.s008.pdf]

# Genomic Diversity of Cluster CE

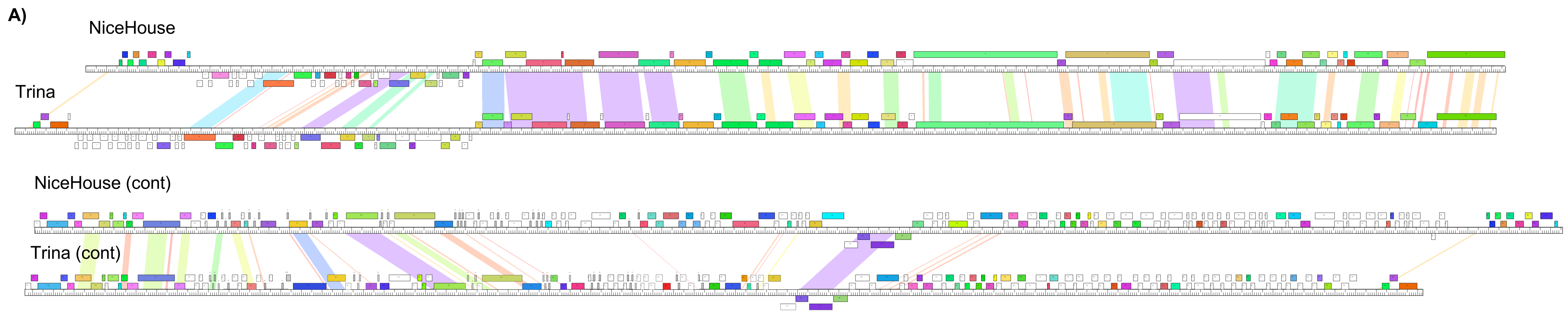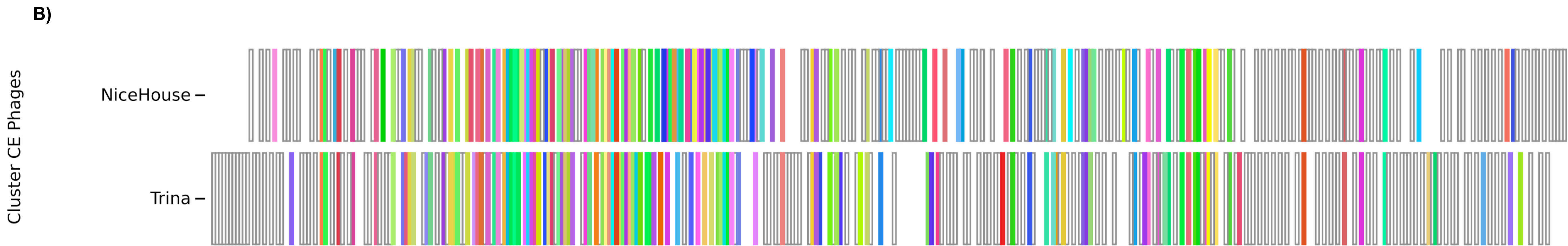

Supplement: S3 Fig — See S1 Fig for details. (PDF) [file pone.0352686.s009.pdf]

# Genomic Diversity of Cluster CF

A)

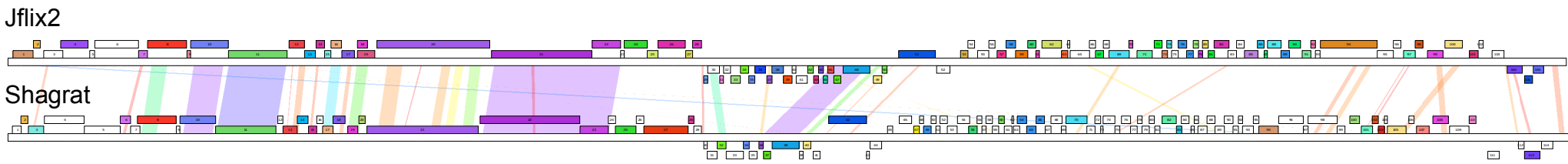

B)

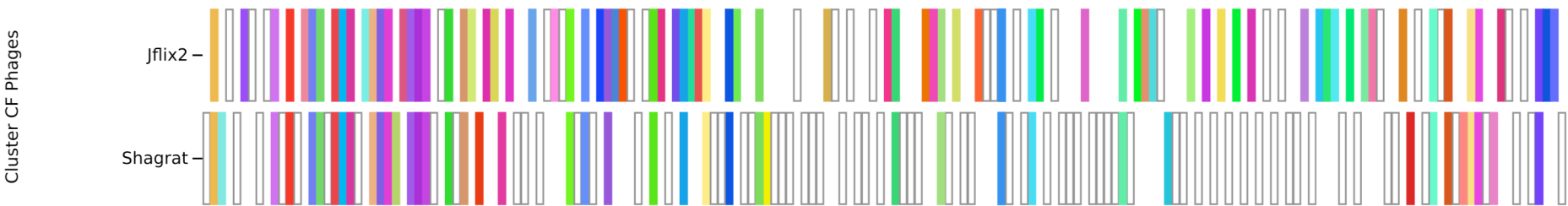

S4\_Figure

Supplement: S4 Fig — See S1 Fig for details. (PDF) [file pone.0352686.s010.pdf]

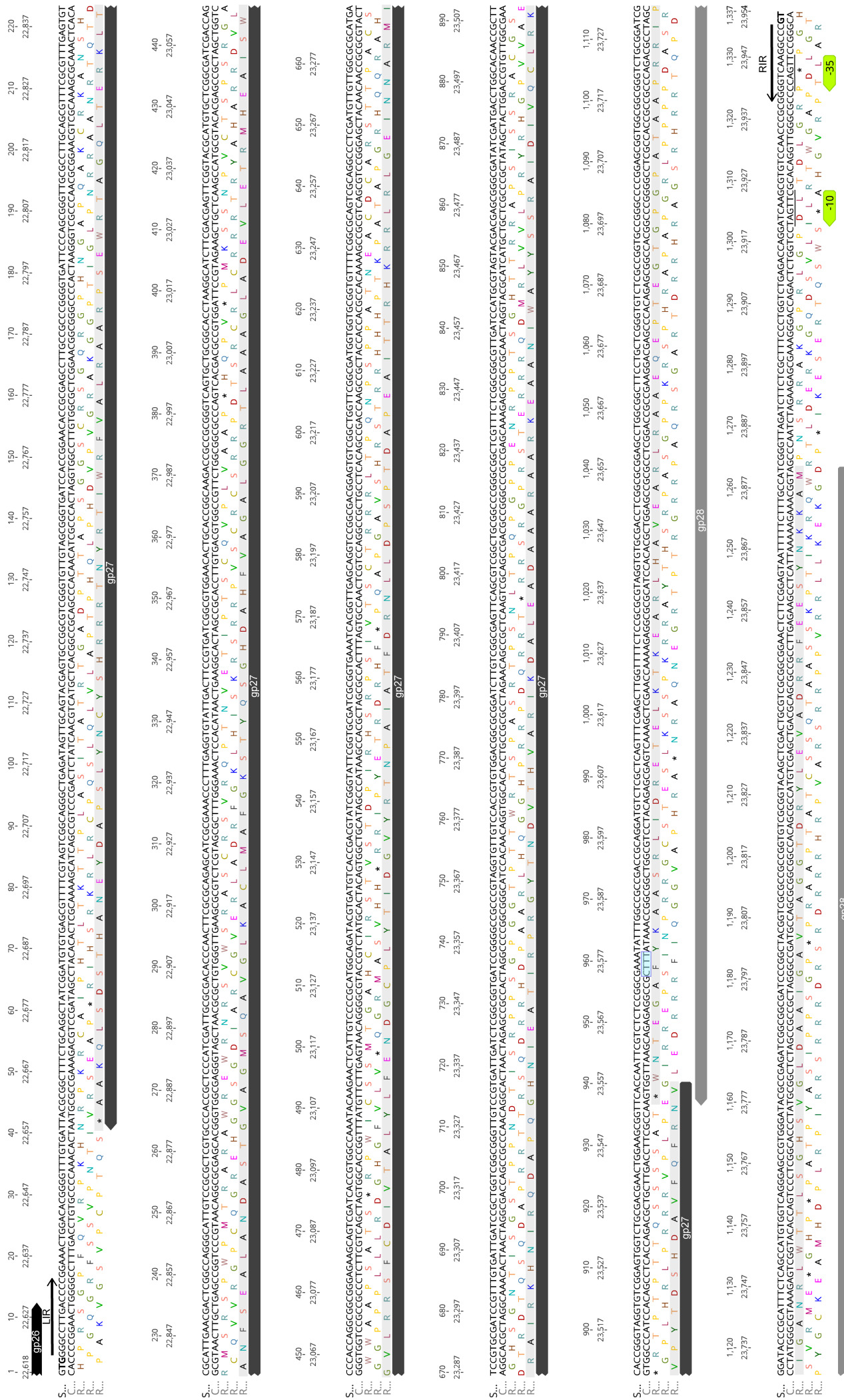

Supplement: S6 Fig — The direct repeats (TG) are indicated in bolded nucleotides and the left and right inverted repeats (LIR and RIR) are indicated by arrows. The gray arrows represent protein coding genes with gp28 (light gray) and gp27 (dark gray) as ORF 1 and ORF2 of the insertion sequence, respectively. The −10 and −35 boxes of a putative promoter overlapping the RIR is indicated by light green arrows and underlined nucleotides. The reading frames for Sleepyhead gp27 and 28 are highlighted in light gray. The TTTC tetramer at the 3’ end of ORF1 (gp28) that potentially signals a −1 programmed ribosomal frameshift is highlighted in light blue. (PDF) [file pone.0352686.s012.pdf]

Sleepyhead (Singleton)

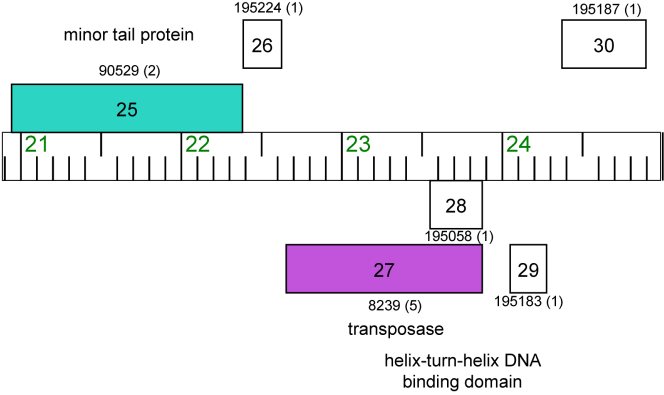

Blueberry (CV)

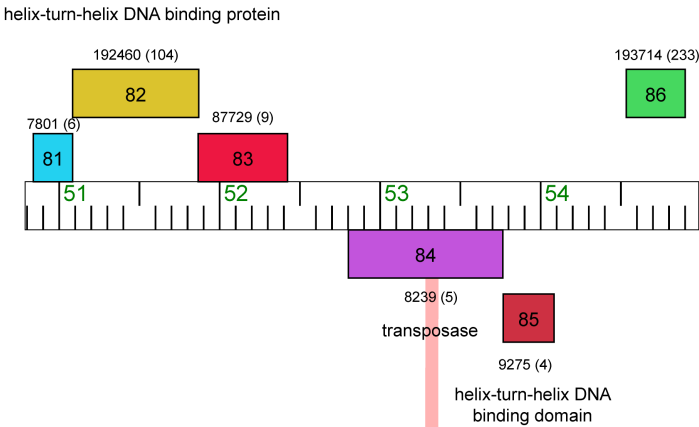

UncleRicky (F1)

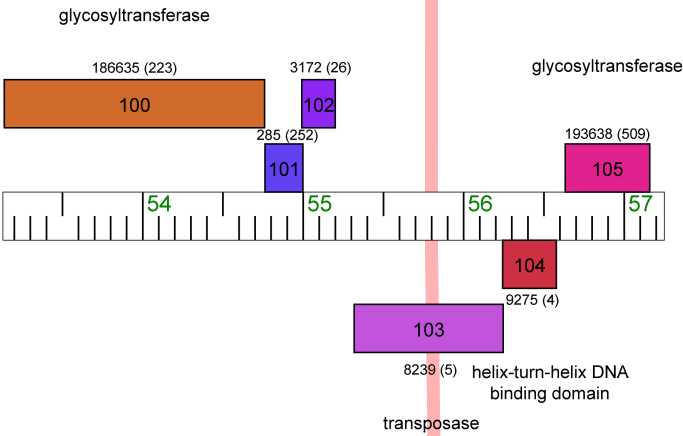

Whitney (DN1)

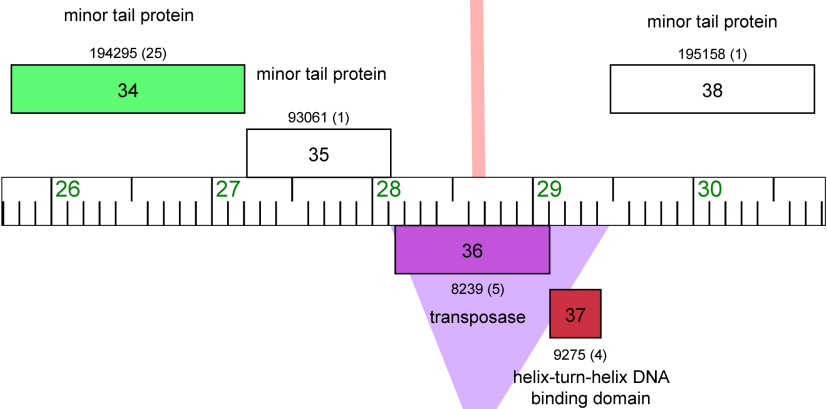

Cucurbita (CQ1)

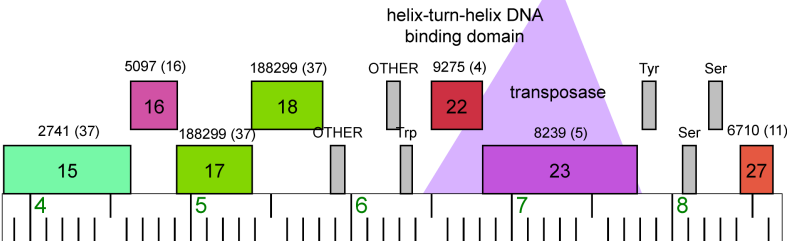

Supplement: S7 Fig — The DDE transposase (Pham 8239; in purple) is found in the genomes of Rhodococcus phage, Sleepyhead (Singleton), Gordonia phages Blueberry (CV), Whitney (DN1), Cucurbita (CQ1) and Mycobacterium phage UncleRicky (F1). Sleepyhead 27 encodes a DDE transposase and gene 28 has strong HHpred alignments to helix-turn-helix DNA binding domains associated with transposases (PF01710) [59]. The DDE transposase pham is found in the phage genomes of UncleRicky (Subcluster F1), Cucurbita (cluster CQ), Blueberry (Cluster CV), and Whitney (Cluster DN1) (Fig 14). The insertion sequence in these phage genomes has a second ORF that belongs to a different pham than Sleepyhead gp28, however all these genes have the same HHpred alignments to transposase associated helix-turn-helix DNA binding domains. While the transposase of some insertion sequences is expressed from a single ORF, transposases of the IS3 family typically exist as two overlapping open reading frames with ORF2 being in the −1 frame relative to ORF1 [38]. These have a −1 programmed ribosomal frameshift in the overlapping region of ORF1 and ORF2 that is signaled by a X-XXZ-ZZN heptamer or a Z-ZZN tetramer [25,60]. A T-TTC tetramer exists just upstream of the Sleepyhead gp28 stop codon that would allow a −1 frameshift and translation of Sleepyhead gp28:27 fusion protein 408 amino acids long (S6 Fig) [61,62]. (PDF) [file pone.0352686.s013.pdf]

Whack (Singleton)

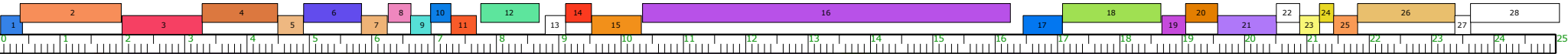

REQ2 (Singleton)

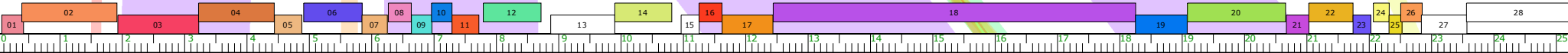

Whack (Singleton)

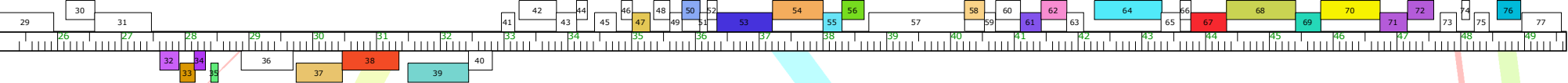

REQ2 (Singleton)

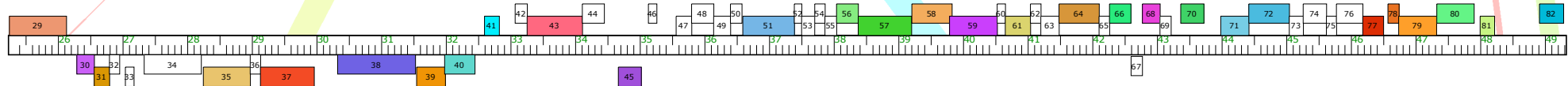

S8 Figure

Supplement: S8 Fig — Phamerator.org map. Each phage genome is shown with predicted genes represented as boxes above or below the genome reflecting rightwards and leftwards transcription, respectively. Each colored box represents a gene, colored according to pham membership as defined by Phamerator.org. White boxes are orphams (genes with no other phamily members). The shading between genomes indicates pairwise nucleotide identity in rainbow order, with purple indicating high similarity, red indicating low similarity, and white indicating no similarity. (PDF) [file pone.0352686.s014.pdf]
