# Supplementary material for: Characterization of genomic diversity in bacteriophages infecting Rhodococcus
Source: PLoS One. 2026 Jun 29;21(6):e0352686. doi: 10.1371/journal.pone.0352686 (PMC13313380; doi:10.1371/journal.pone.0352686)
Supplement: S5 Fig — See S1 Fig for details of A) and B). C). Display of Gene Content Similarities (GCS) amongst the Rhodococcus Cluster CR phages and singleton ChewyVIII. (PDF) [file pone.0352686.s011.pdf]

Genomic diversity of Cluster CR phages

A)

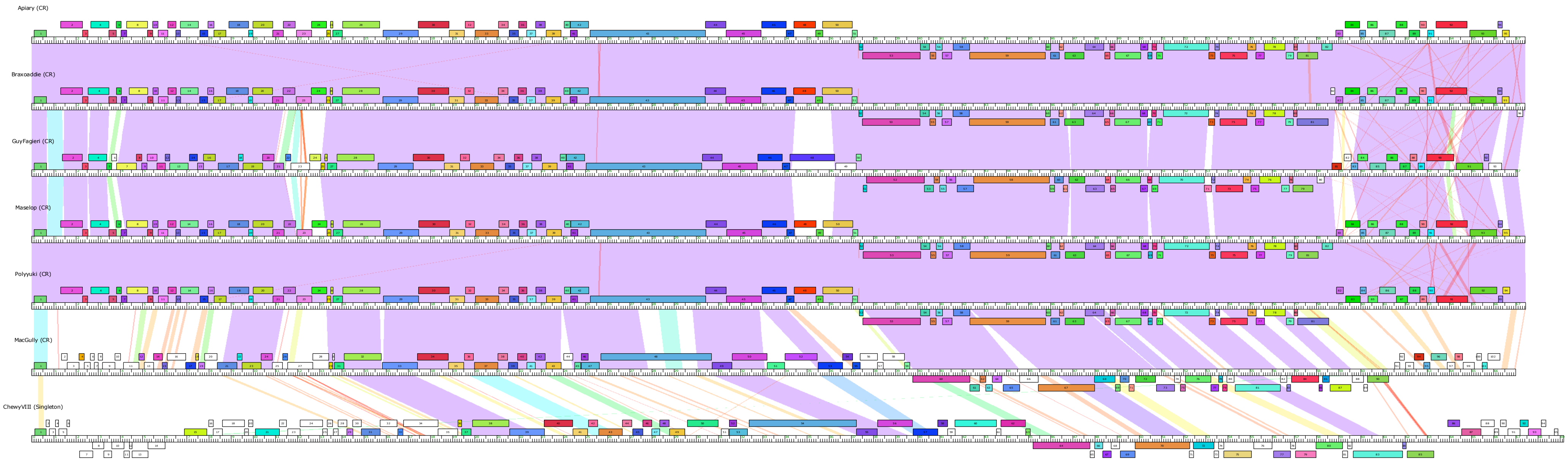

B)

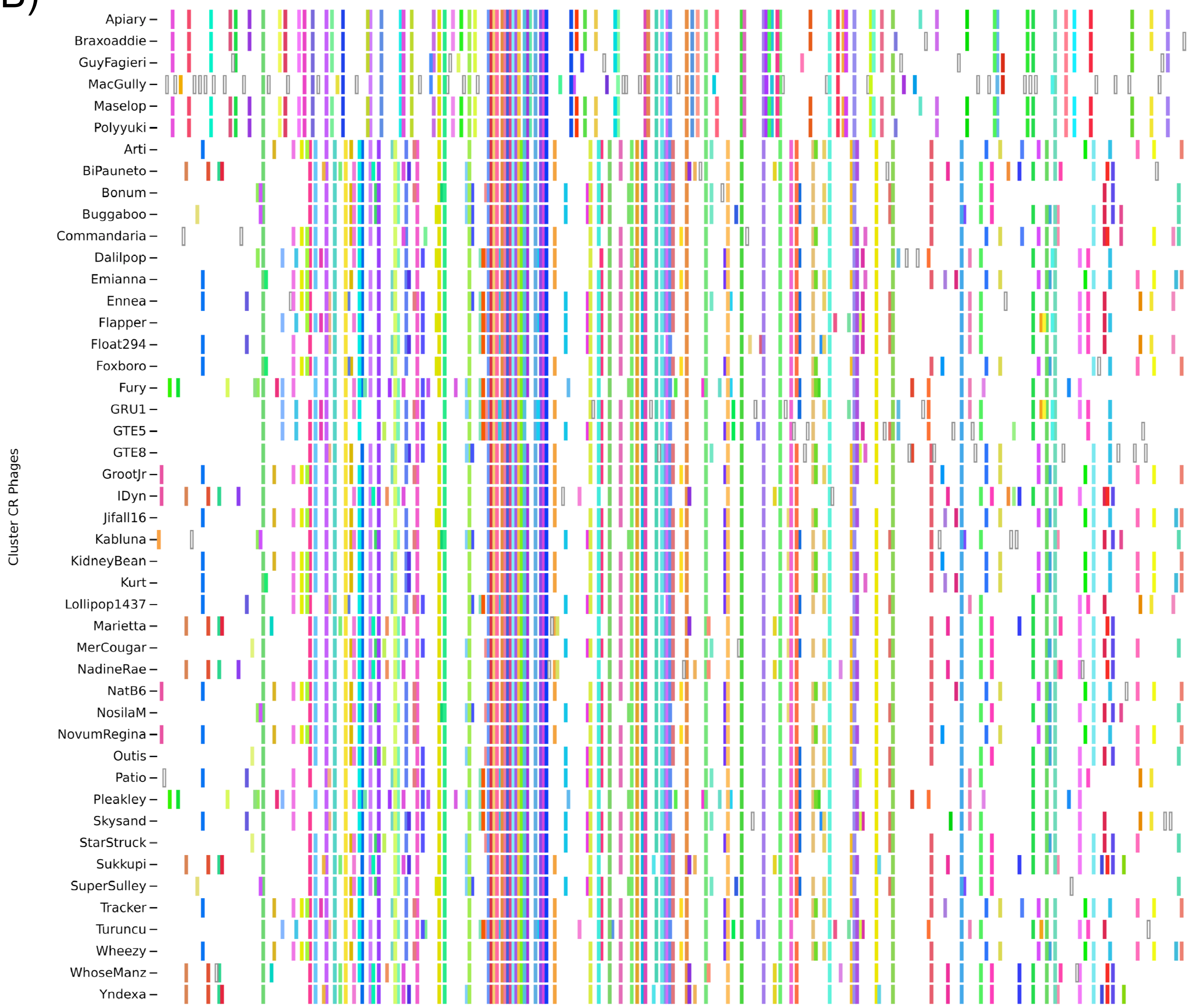

C)

Phage	Apiary (CR6)	GuyFagieri (CR6)	Maselop (CR6)	Polyyuki (CR6)	MacGully (CR7)	ChewyVIII (Singleton)
**Braxoaddie (CR6)**	97.4	85.7	97.4	99.0	56.6	32.5

S5_Figure
